# New Constructions of Identity-Based Dual Receiver Encryption from Lattices

**DOI:** 10.3390/e22060599

**Published:** 2020-05-28

**Authors:** Yuan Liu, Licheng Wang, Xiaoying Shen, Lixiang Li

**Affiliations:** State Key Laboratory of Networking and Switching Technology, Beijing University of Posts and Telecommunications, Beijing 100876, China; yuanl_1011@163.com (Y.L.); shenxiaoying@bupt.edu.cn (X.S.); lixiang@bupt.edu.cn (L.L.)

**Keywords:** lattice, dual receiver encryption, identity-based cryptography, learning with errors, adaptively secure

## Abstract

Dual receiver encryption (DRE), being originally conceived at CCS 2004 as a proof technique, enables a ciphertext to be decrypted to the same plaintext by two different but dual receivers and becomes popular recently due to itself useful application potentials such secure outsourcing, trusted third party supervising, client puzzling, etc. Identity-based DRE (IB-DRE) further combines the bilateral advantages/facilities of DRE and identity-based encryption (IBE). Most previous constructions of IB-DRE are based on bilinear pairings, and thus suffers from known quantum algorithmic attacks. It is interesting to build IB-DRE schemes based on the well-known post quantum platforms, such as lattices. At ACISP 2018, Zhang et al. gave the first lattice-based construction of IB-DRE, and the main part of the public parameter in this scheme consists of 2n+2 matrices where *n* is the bit-length of arbitrary identity. In this paper, by introducing an injective map and a homomorphic computation technique due to Yamada at EUROCRYPT 2016, we propose another lattice-based construction of IB-DRE in an even efficient manner: The main part of the public parameters consists only of $2pn^{\frac{1}{p}}+2$ matrices of the same dimensions, where p(≥2) is a flexible constant. The larger the *p* and *n*, the more observable of our proposal. Typically, when p=2 and n=284 according to the suggestion given by Peikert et al., the size of public parameters in our proposal is reduced to merely 12% of Zhang et al.’s method. In addition, to lighten the pressure of key generation center, we extend our lattice-based IB-DRE scheme to hierarchical scenario. Finally, both the IB-DRE scheme and the HIB-DRE scheme are proved to be indistinguishable against adaptively chosen identity and plaintext attacks (IND-ID-CPA).

## 1. Introduction

With the rapid development of the internet of things, more and more user tend to encrypt their data and then outsource their data to the cloud server. These outsourced data may contain some sensitive information such as financial, medical data, national security-related data, etc. Therefore, a reliable third party or government department is required to supervise these data, and if it is necessary, the regulator can decrypt the ciphertext and view the plaintext information of these data. Dual receiver encryption (DRE) [1] allows that a ciphertext can be decrypted to the same plaintext by two independent receivers. For the above scenario, DRE is a good handy tool. It not only guarantees the encrypted transmission and storage of data, but also enables data to be supervised by a reliable third party. In addition, DRE also can form a joint program with other cryptographic scheme. In [1], Diament et al. combined a DRE scheme with a signature scheme to achieve that a user can use a same public/secret key pair to complete the encryption and signature functions. In 2014, Chow et al. [2] proposed a DRE-PKE joint scheme and appended some stable properties to the DRE and made it more practical in the construction of plaintext-awareness public key encryption. Furthermore, DRE also can be used to construct a client puzzles mechanism based on decryption. Design client puzzles mechanism between the clients and severs can prevent servers from suffering from resource-depletion attacks. Diament et al. pointed out in [1] that the client puzzles mechanism can be applied to secure transport protocol, e.g., TLS. The DRE cryptographic primitive can be easily construct a deniable authentication system [3]. As a special kind of PKE, DRE also face the general certificate management problem. To solve the problem of certificate management in the traditional PKE, Zhang et al. [4] gave an identity-based variant version of DRE, named identity-based dual receiver encryption (IB-DRE). In this scheme, they constructed two IB-DRE schemes based on the identity-based encryption (IBE) scheme in [5].

However, all the above (IB)-DRE schemes are based on bilinear pairing groups. Since Shor [6] proposed a polynomial time quantum algorithm in 1997 which can solve discrete logarithm problem (DLP) and prime factorization. This type of bilinear pairing groups based schemes are not secure and can not resist the quantum attack. Since then, scholars have begun to study post-quantum cryptography (PQC). Lattices-based cryptography is a research hotspot for PQC. It has the following properties: high efficiency, simplicity, parallelization and average case/worst case equivalence property. In 1997, Ajtai and Dwork [7] first constructed a public key encryption scheme by using the problem on lattices. This scheme relies on the worst-case hardness of uSVP [8], and the key and ciphertext size is too large. Until 2005, Regev [9] presented another public key encryption scheme which security based on the learning with errors (LWE) problem. He proved that a LWE-based public key encryption can resist quantum attack. Then the researchers begin to study LWE-based public key encryption scheme. Based on LWE, many public key encryption schemes have been proposed, such as LWE-based IBE schemes [10,11,12,13,14], LWE-based attribute-based encryption schemes [15,16,17,18,19]. To our knowledge, the first lattice-based IB-DRE scheme was proposed in 2018 [20] (Next we use how Zhang18 denotes it). The public parameters size in this scheme consists of 2n+2 matrices which lead a high storage cost and communication cost. Additionally, when some users apply for their privacy key to the key generation center (KGC) at the same time, KGC may face great system pressure. Therefore, in order to reduce the storage cost, it is meaningful to construct a lattice-based IB-DRE with short public parameters and then extend it a hierarchical scenario to reduce the stress of KGC.

OUR CONTRIBUTION. In this paper, we firstly propose a new lattice-based construction of IB-DRE scheme which can resist quantum attack. By using a homomorphic computation technique and an injective map function, comparing to the first lattice-based IB-DRE [20], we reduce the public parameters size from 2n+2 matrices to $2pn^{\frac{1}{p}}+2$ matrices where *n* is the bit-length of arbitrary identity and p(≥2) is a flexible constant. By choosing appropriate *p* and *n*, the pp size can be reduced by almost at least 88% compared to Zhang18. In addition, considering the hierarchical scenario, we extend it to a hierarchical IB-DRE (HIB-DRE), which is not considered in Zhang18. A HIB-DRE can reduce the stress of the key generation center (KGC). The public parameters size of the HIB-DRE is also reduced from 2dn+2 matrices to $2dpn^{\frac{1}{p}}+2$ matrices where *d* is the maximum hierarchy depth. Finally, our lattice-based IB-DRE scheme and HIB-DRE scheme are proved to be indistinguishable against adaptively chosen identity and plaintext attacks (IND-ID-CPA) in the standard model. Additionally, to improve the encryption efficiency, our two schemes also can convert to a multi-bit encryption scheme by using the same method in [11].

## 2. Preliminarise

**Notation.** We use lowercase black italic alphabet for vectors, as in u, uppercase black italic alphabet for matrices, as in A. [n] denotes a integer set {1,2,⋯,n}. $Z_{q}$ denotes an integer set of mod *q* residue class. $u\in Z_{q}^{n}$ is a *n*-dimension column vector. A n×m matrix is denoted by $A\in Z_{q}^{n\times m}$ where $A=(a_{1},a_{2},\cdots ,a_{m})$. ∥A∥ denotes the $\ell_{2}$-norm length of the longest column of A. $\tilde{A}$ denotes the Gram–Schmidt orthogonalization of the vectors $a_{1},\cdots ,a_{m}$. We refer to $∥\tilde{A}∥$ as the Gram–Schmidt norm of A.

### 2.1. Integer Lattice

**Definition** **1** (Lattices). $b_{1},b_{2},\ldots ,b_{n}\in R^{m}$*are n linearly independent vectors, and the lattice* Λ *generated by the following formula:*

$$\Lambda =L\left(B\right)=\{\sum_{i=1}^{n}x_{i}b_{i}:x_{i}\in Z,\left(i=1,\cdots ,n\right)\}.$$

*Note that*$B=[b_{1},b_{2},\cdots ,b_{n}]$*is a basis of* Λ*, n is the rank and m is the dimension.*

**Definition** **2** (Integer lattices). *For prime* $q,A\in Z_{q}^{n\times m}$ *, and* $u\in Z_{q}^{n}$ *, define:*

$$\Lambda_{q}\left(A\right)=\left\{y\in Z^{m}s.t.\exists s\in Z_{q}^{n},A^{⊤}s=y\left(\;\mathrm{mod}\;q\right)\right\}.$$

$$\Lambda_{q}^{⊥}\left(A\right)=\left\{y\in Z^{m}s.t.Ay=0\left(\mathrm{mod}q\right)\right\}.$$

$$\Lambda_{q}^{u}\left(A\right)=\left\{y\in Z^{m}s.t.Ay=u\left(\mathrm{mod}q\right)\right\}.$$

### 2.2. Discrete Gaussians

**Definition** **3** (Discrete Gaussian). *For a positive integer* s∈R *and a vector* $c\in R^{m}$ *, we defined a Gaussian distribution with center* c *and variance s as follow:*

$$D_{\Lambda ,\sigma ,c}=\frac{\rho_{\sigma ,c}\left(x\right)}{\rho_{\sigma ,c}\left(\Lambda \right)}=\frac{\rho_{\sigma ,c}\left(x\right)}{\sum_{x\in \Lambda }\rho_{\sigma ,c}\left(x\right)}$$

*where* σ>0 *is a parameter, and* $\rho_{\sigma ,c}\left(x\right)=\exp\left(-\pi \frac{{∥x-c∥}^{2}}{\sigma^{2}}\right)$ *.*

**Lemma** **1** ([10]). *Let* q≥2*,* $A\in Z_{q}^{n\times m}$ *with* m>n*,* $T_{A}\in Z_{q}^{m\times m}$ *be a basis for* $\Lambda_{q}^{⊥}\left(A\right)$ *and* $\sigma \geq ∥\tilde{T_{A}}∥\omega \left(\sqrt{\log m}\right)$*. Then for* $c\in R^{m}$ *and* $u\in Z_{q}^{n}$*, we have:*

*(1).*  $\mathrm{Pr}[e∼D_{\Lambda_{q}^{u}\left(A\right),\sigma }:∥e∥>\sigma \sqrt{m}]\leq negl\left(n\right)$*.*
*(2).*  *There is a probabilistic polynomial-time (PPT) algorithm SampleGaussian(*A*,*$T_{A}$*, σ,*c*) that outputs a vector*$e\in \Lambda_{q}^{⊥}\left(A\right)$*drawn from a distribution statistically close to*$D_{\Lambda ,\sigma ,c}$*.*
*(3).*  *There is a PPT algorithm SamplePre(*A*,*$T_{A}$*,*u*,*c*) that outputs a vector*$e\in \Lambda_{q}^{u}\left(A\right)$*sampled from a distribution statistically close to*$D_{\Lambda_{q}^{u}\left(A\right),\sigma }$*.*

### 2.3. Related Algorithms

For any integer q,n,m and *q* is a prime, there are PPT algorithms such that:

(1). TrapGen(q,n) ([21]): outputs a pair matrices $A\in Z_{q}^{n\times m}$ and $T_{A}\in Z_{q}^{m\times m}$ where $T_{A}$ is a basis for $\Lambda_{q}^{⊥}\left(A\right)$ and m≥3(1+δ)nlogq for some δ>0.
(2). SampleLeft(A, $M_{1}$, $T_{A}$, u, σ) ([11]): given $A\in Z_{q}^{n\times m}$, $M_{1}\in Z_{q}^{n\times m_{1}}$, a basis $T_{A}\in Z_{q}^{m\times m}$ for $\Lambda_{q}^{⊥}\left(A\right)$, $u\in Z_{q}^{n}$ and a Gaussian parameter $\sigma >∥\tilde{T_{A}}∥\omega \left(\sqrt{\log(m+m_{1})}\right)$, outputs a vector $e\in Z^{m+m_{1}}$ and the vector e is not statistically distinguishable from $D_{\Lambda_{q}^{u}\left(F_{1}\right),\sigma }$ where $F_{1}=\left[A|M_{1}\right]$ and $F_{1}\cdot e=u\left(\;\mathrm{mod}\;q\right)$.
(3). SampleRight(A, B, R, $T_{B}$, u, σ) ([11]): given $A\in Z_{q}^{n\times k}$, $B\in Z_{q}^{n\times m}$, $R\in Z_{q}^{k\times m}$, a basis $T_{B}$ for $\Lambda_{q}^{⊥}\left(B\right)$, $u\in Z_{q}^{n}$ and a Gaussian parameter $\sigma >∥\tilde{T_{B}}∥s_{R}\omega \left(\sqrt{\log m}\right)$ where $s_{R}=∥\tilde{R}∥<O\left(\sqrt{m}\right)$, outputs a vector $e\in Z^{m+k}$ and the vector e is not statistically distinguishable from $D_{\Lambda_{q}^{u}\left(F_{2}\right),\sigma }$ where $F_{1}=\left[A|AR+B\right]$ and $F_{2}\cdot e=u\left(\mathrm{mod}q\right)$.
  Note that in our scheme, we let B=yG where $y\in Z_{q}$ and y≠0. Then taking $T_{G}$ as the input basis for the lattices $\Lambda_{q}^{⊥}\left(G\right)$.

**Lemma** **2** ([22]). *For any integer $q\geq 2,n\geq 1,w=nt,t=\lceil \log_{2}q\rceil$, there is a gadget matrix $G\in Z_{q}^{n\times w}$ such that:*

1. The lattice $\Lambda_{q}^{⊥}\left(G\right)$ has a known basis $T_{G}$ where $∥\tilde{T_{G}}∥\leq \sqrt{5}$.
2. There is a PPT algorithm $G^{-1}$ that takes input a vector $u\in Z_{q}^{n}$ and output a vector $x=G^{-1}\left(u\right)$ where $x\in {\left\{0,1\right\}}^{w}$ and Gx=u. Note that $G^{-1}$ is a function, not a matrix.

### 2.4. Homomorphic Computation

The ideal of homomorphic trapdoor computation is introduced in [14].

Let *p* be a positive integer, it has a function Eval: ${\left(Z_{q}^{n\times m}\right)}^{p}\to Z_{q}^{n\times m}$ which inputs *p* matrices $A_{1}$, $A_{2}$,⋯,$A_{p}$ and outputs a matrix.

$${\mathrm{Eval}}_{p}\left(A_{1},A_{2},\cdots ,A_{p}\right)=\left\{\begin{array}{cc} A_{1} & p=1\\ A_{1}\cdot G^{-1}\left({\mathrm{Eval}}_{p-1}\left(A_{2},\cdots ,A_{p}\right)\right) & p\geq 2 \end{array}\right.$$

Here $G^{-1}$ is a deterministic function that has the following feature:

$$\begin{array}{cc} G^{-1}:\; & Z_{q}^{n\times m}\to {\left(0,1\right)}^{m\times m}\\ & U\to X,s.t.GX=U \end{array}$$

**Lemma** **3** ([14]). *Let* $A,A_{1},\cdots ,A_{p}\in Z_{q}^{n\times m}$ *and* $R_{1},\cdots ,R_{p}\in Z^{m\times m}$*, for* i∈[p]*, it has* $A_{i}=AR_{i}+y_{i}G$*. In addition, assume that* $∥R_{i}∥\leq m$*,* $|y_{i}|\leq \delta$ *and* δ>m*, there exists* TrapEval *algorithm that takeing* $R_{1},\cdots ,R_{p},y_{1},\cdots ,y_{p}$ *as input and outputs a matrix* $R^{{}^{\prime }}$ *such that* ${Eval}_{p}\left(A_{1},A_{2},\cdots ,A_{p}\right)=AR^{{}^{\prime }}+y_{1}\cdots y_{p}\cdot G$ *and* $∥R^{{}^{\prime }}∥\leq mp\delta^{p-1}$*.*

### 2.5. LWE Hardness Assumption

**Definition** **4.** *Give a prime q, a positive integer n and a distribution ${\bar{\Psi }}_{\alpha }$ over $Z_{q}$. A $\left(Z_{q},n,{\bar{\Psi }}_{\alpha }\right)$-LWE problem instance consists of access to an unspecified challenge oracle O, being either a truly random sampler $O_{s}^{{}^{\prime }}$ or a noisy pseudo-random sampler $O_{s}$ carrying some constant random secret key $s\in Z_{q}$, whose behaviors are as follows, respectively:*

*$O_{s}$: Outputs samples of the form $\left(w_{i},v_{i}\right)=\left(w_{i},w_{i}^{⊤}s+\chi_{i}\right)$$\in Z_{q}^{n}\times Z_{q}$, where $s\in Z_{q}^{n}$ is a uniformly distributed secret key, $\chi_{i}$ is a noise component from ${\bar{\Psi }}_{\alpha_{i}}$, and $w_{i}$ is uniform in $Z_{q}^{n}$.*

*$O_{s}^{{}^{\prime }}$: Outputs truly uniform random samples $\left(w_{i},v_{i}\right)$ from $Z_{q}^{n}\times Z_{q}$.*

*The $\left(Z_{q},n,{\bar{\Psi }}_{\alpha }\right)$-LWE problem allows a number of queries to the challenge oracle O. We say an algorithm A decides a $\left(Z_{q},n,{\bar{\Psi }}_{\alpha }\right)$-LWE problem if $\mathrm{LWE}Adv\left[A\right]=|\mathrm{Pr}\left[A^{O_{s}}=1\right]-\mathrm{Pr}\left[A^{O_{s}^{{}^{\prime }}}=1\right]|$ is non-negligible for a random $s\in Z_{q}^{n}$.*

**Theorem** **1** ([9]). *If there exists an efficient, possibly quantum, algorithm for deciding the $\left(Z_{q},n,{\bar{\Psi }}_{\alpha }\right)$-LWE problem for $q>2\sqrt{n}/\alpha$ then there is an efficient quantum algorithm for approximating the SIVP and GapSVP problems to within $\tilde{O}\left(n/\alpha \right)$ factors in the $\ell_{2}$ norm, in the worst case.*

**Lemma** **4** ([10]). *Let e be some vector in $Z^{m}$ and let $y\overset{R}{\leftarrow }{\bar{\Psi }}_{\alpha }^{m}$. Then the quality $|e^{⊤}y|$ treated as an integer in [0,q−1] satisfies:*

(1) $$|e^{⊤}y|\leq ∥e∥q\alpha \omega \left(\sqrt{\log m}\right)+∥e∥\sqrt{m}/2$$

*with all but negligible probability in m.*

As a special case, Lemma 4 shows that if $x\overset{R}{\leftarrow }{\bar{\Psi }}_{\alpha }$ is treated as an integer in [0,q−1] satisfies

(2) $$|x|\leq q\alpha \omega (\sqrt{\log m})+1/2$$

with all but negligible probability in *m*.

### 2.6. Three Basic Lemmas

**Lemma** **5** ([11]). *For a random matrix $R\in {\left\{-1,1\right\}}^{k\times m}$, there is a universal constant C such that:*

(3) $$\mathrm{Pr}[∥R∥>C\sqrt{k+m}]<e^{-(k+m)}.$$

**Lemma** **6** (Leftover Hash Lemma [11]). *For m>(n+1)logq+ω(logn) and a prime q>2. Let $R\in {\left\{-1,1\right\}}^{m\times k}$, $A\in Z_{q}^{n\times m}$ and $B\in Z_{q}^{n\times k}$ be uniformly random matrices. Then the distribution (A, AR, $R^{⊤}w$) is negl(n)-close to the distribution (A, B, $R^{⊤}w$).*

**Lemma** **7** (Smudging out Lemma [14]). *Let $x_{0}\in Z^{m}$ be a fixed vector and $∥x_{0}{∥}_{\infty }\leq \delta$. $x\leftarrow {\left\{-B,B\right\}}^{m}$ is a uniformly random vector. Then the two distributions $x_{0}$ and $x_{0}+x$ are within statistical distance mδ/B.*

### 2.7. Definitions of (H)IB-DRE and Adaptive-ID Security Model

Identity-based dual receiver encryption (IB-DRE) enables a ciphertext to be decrypted to the same plaintext by two different receivers since it embeds two independent user’s identity in the encrypt phase. Considering the definition of IB-DRE in [20]. We give the following definition of IB-DRE. An IB-DRE scheme consists the following four algorithms.

(1). $\mathrm{Setup}(1^{n}$)→ (pp,msk): on input the security parameter $1^{n}$. This algorithm outputs the public parameters pp and master secret key msk.
(2). KeyGen(pp,id,msk)$\to sk_{\mathrm{id}}$: On input the public parameter pp, a user’s identity id and the master secret key msk. This algorithm KeyGen outputs the secret key $sk_{\mathrm{id}}$. In the scheme, we let ${\mathrm{id}}_{1}$, ${\mathrm{id}}_{2}$ denote the first receiver and the second receiver respectively.
(3). $\mathrm{Encrypt}(pp,{\mathrm{id}}_{1},{\mathrm{id}}_{2},\mu$)→c: on input the public parameter pp, the user’s identities ${\mathrm{id}}_{1},{\mathrm{id}}_{2}$ and the message bit μ∈{0,1}. This algorithm outputs the ciphertext c.
(4). $\mathrm{Decrypt}(pp,sk_{{\mathrm{id}}_{i^{\prime }}},c$)→μ: on input the public parameter pp, a user’s secret key ${\left(sk_{{\mathrm{id}}_{i^{\prime }}}\right)}_{i^{\prime }\in \left\{1,2\right\}}$, and the ciphertext c. This algorithm outputs a message μ.

The definition of IB-DRE can be easily extended to a hierarchical IB-DRE by following the method in [11].

**Correctness.** For all identities ${\mathrm{id}}_{i^{\prime }}$, all message μ and the ciphertext c←$\mathrm{Encrypt}(pp,{\mathrm{id}}_{1},{\mathrm{id}}_{2},\mu$), we have Pr[Decrypt(pp$,sk_{{\mathrm{id}}_{1}},$***c***)=Decrypt(pp$,sk_{{\mathrm{id}}_{2}},c$)=μ]=1−negl(n).

The definition of adaptive-ID security model is adapted from [11]. It can be described by a IND-ID-CPA game between a challenger B and an adversary A as follows:

**Setup**. The challenger B runs the $\mathrm{Setup}(1^{n}$) algorithm to generate the public parameters pp and the master key msk, and send pp to A.

**Phase 1**. The adversary A makes secret key queries for different identities adaptively.

**Challenge**. The adversary A sends a message bit $\mu^{*}\in \left\{0,1\right\}$ and the target identities $\left({\mathrm{id}}_{1}^{*},{\mathrm{id}}_{2}^{*}\right)$ to B, and the target identities $\left({\mathrm{id}}_{1}^{*},{\mathrm{id}}_{2}^{*}\right)$ should not be asked in **Phase 1**. The challenger B randomly chooses r∈{0,1} and a randomly ciphertext space C. If r=0, it send the challenge ciphertext $c^{*}$=Encrypt($pp,{\mathrm{id}}_{1}^{*},{\mathrm{id}}_{2}^{*},\mu^{*}$) to A. If r=1, it send a randomly challenge ciphertext $c^{*}\in C$ to A.

**Phase 2**. The adversary A also makes secret key queries for different identities adaptively as **Phase 1**. It can not ask for $\left({\mathrm{id}}_{1}^{*},{\mathrm{id}}_{2}^{*}\right)$.

**Guess**. The adversary A outputs its guess $r^{\prime }\in \left\{0,1\right\}$ and wins if $r^{\prime }=r$. We define the advantage of the adversary A in attacking IB-DRE scheme as $\varepsilon =|\mathrm{Pr}\left[r^{\prime }=r\right]-\frac{1}{2}|$.

## 3. Adaptively Secure IB-DRE Scheme with Short Public Parameters

As we all know, in the adaptively secure IBE scheme in [11], for an identity $\mathrm{id}=\left(b_{1},b_{2},\cdots ,b_{n}\right)\in {\left\{0,1\right\}}^{n}$, the key generation matrix/encryption matrix is $F_{\mathrm{id}}=\left[A_{0}|B_{0}+\sum_{i=1}^{n}b_{i}A_{i}\right]$ where ${\left(A_{i}\right)}_{i\in [n]}$ is the matrices in the public parameters. Thus, if we want to construct an adaptively secure identity based dual receiver encryption (IB-DRE) scheme, the public parameters will be 2n+2 matrices which lead to high storage cost.

In this section, we propose an adaptively secure IB-DRE scheme with short public parameters. There are four algorithms in this scheme: Setup, KeyGen, Encrypt and Decrypt. The main method to reduce the public parameters is that in the Setup phase we introduce an injective map function which can map a *n*-bits identity to a subset of ${\left[1,l\right]}^{p}$, and here we let $l=\lceil n^{\frac{1}{p}}\rceil$. Additionally, we also introduce a homomorphic computation technique to ensure that our scheme achieves a strong secure notion, i.e., indistinguishability of ciphertext under the adaptive chosen-identity chosen-plaintext attack (IND-ID-CPA). For the key generation matrix/encryption matrix, let $H\left(\mathrm{id}\right)=B_{0}+\sum_{i=1}^{n}b_{i}A_{i}$, then $F_{\mathrm{id}}=\left[A_{0}|H\left(\mathrm{id}\right)\right]$. In the KeyGen phase, we use the same SampleLeft algorithm in [11] to generated the two independent users’ secret keys but change the way H(id) is generated. H(id) is computed by a function Eval: ${\left(Z_{q}^{n\times m}\right)}^{p}\to Z_{q}^{n\times m}$ of the public parameters where function Eval is a part of homomorphic computation technique. When encrypting a message bit, it should use two independent receivers’ public keys to encrypt the message. Then the ciphertext can be decrypted to the same message by the two independent receivers.

By doing this, we reduce the size of public parameters from 2n+2 (i.e., O(n)) matrices to $2pn^{\frac{1}{p}}+2$ (i.e., $O(n^{\frac{1}{p}})$) matrices where *p* is a flexible constant and can affect the reduction cost. Next we will describe our scheme step by step.

### 3.1. Our Construction

The adaptively secure IB-DRE scheme with short public parameters consists the following four algorithms.

(1). $\mathrm{Setup}(1^{n}$)→ (pp,msk): on input the security parameter $1^{n}$. This algorithm outputs the public parameters pp and master secret key msk, do:
  - Perform algorithm TrapGen to generate a uniformly matrix $A_{0}\in Z_{q}^{n\times m}$ and a trapdoor $T_{A_{0}}\in Z^{m\times m}$.
  - For an identity $\mathrm{id}=\left(b_{1},b_{2},\cdots ,b_{n}\right)\in {\left\{0,1\right\}}^{n}$, select an injective map F that maps an identity to a subset F(id) of ${\left[1,l\right]}^{p}$ where $l=\lceil n^{\frac{1}{p}}\rceil$.
  - For (i,j)∈[p,l], select 2pl matrices $A_{i,j},A_{i,j}^{{}^{\prime }}$.
  - Select a uniformly random matrices $B_{0}$ and a uniformly random vector $u\in Z_{q}^{n}$.
  The public parameter $pp=\{A_{0},{\left(A_{i,j},A_{i,j}^{{}^{\prime }}\right)}_{\left(i,j)\in [p,l\right]},B_{0},u\}$, the master secret key $msk=\{T_{A_{0}}\}$.
  Recall that by the function ${\mathrm{Eval}}_{p}$: ${\left(Z_{q}^{n\times m}\right)}^{p}\to Z_{q}^{n\times m}$, for the two identities $F\left({\mathrm{id}}_{1}\right),F\left({\mathrm{id}}_{2}\right)$, we have a deterministic function H such that
  $$H\left({\mathrm{id}}_{1}\right)=B_{0}+\sum_{\left(j_{1},j_{2},\cdots ,j_{p}\right)\in F\left({\mathrm{id}}_{1}\right)}{\mathrm{Eval}}_{p}\left(A_{1,j_{1}},A_{2,j_{2}},\cdots ,A_{p,j_{p}}\right).$$
  $$H\left({\mathrm{id}}_{2}\right)=B_{0}+\sum_{\left(j_{1},j_{2},\cdots ,j_{p}\right)\in F\left({\mathrm{id}}_{2}\right)}{\mathrm{Eval}}_{p}\left(A_{1,j_{1}}^{{}^{\prime }},A_{2,j_{2}}^{{}^{\prime }},\cdots ,A_{p,j_{p}}^{{}^{\prime }}\right).$$
(2). KeyGen(pp,id,msk)$\to sk_{\mathrm{id}}$: On input the public parameter pp, the user’s identity id and the master secret key msk. This algorithm KeyGen outputs the secret key $sk_{\mathrm{id}}$, it works as follows:
  - It runs algorithm $\mathrm{SampleLeft}(A_{0},H\left(\mathrm{id}\right),T_{A_{0}})$ to generate e such that $F_{\mathrm{id}}\cdot e=u$ where $F_{\mathrm{id}}=\left[A_{0}|H\left(\mathrm{id}\right)\right]$. Then it set $sk_{\mathrm{id}}=e\in Z_{q}^{2m}$.
  - For two independent receivers, we let $e_{1}$ and $e_{2}$ denote the first and second receiver’s secret key.
  The two independent receivers’ secret keys are $sk_{{\mathrm{id}}_{1}}=e_{1}\in Z_{q}^{2m}$, $sk_{{\mathrm{id}}_{2}}=e_{2}\in Z_{q}^{2m}$.
(3). $\mathrm{Encrypt}（pp,{\mathrm{id}}_{1},{\mathrm{id}}_{2},\mu$)→c: On input the public parameter pp, the user’s identities ${\mathrm{id}}_{1},{\mathrm{id}}_{2}$ and the message bit μ∈{0,1}. This algorithm outputs the ciphertext c. it works as follows:
  - It firstly gets $H({\mathrm{id}}_{1})$ and $H({\mathrm{id}}_{2})$ as above.
  - Choose a randomly uniform vector $s\in Z_{q}^{n}$, and error terms $x\overset{{\bar{\Psi }}_{\alpha }}{\leftarrow }Z_{q}$, $x_{1,1},x_{1,2},\overset{{\bar{\Psi }}_{\alpha }^{m}}{\leftarrow }Z_{q}^{m}$, and $x_{2,1},x_{2,2}\leftarrow {\left\{-B,B\right\}}^{m}$, compute
    $$c_{0}=u^{⊤}s+x+\mu \left\lfloor q/2\right\rfloor \in Z_{q}.$$
    $$c_{1}=F_{{\mathrm{id}}_{1}}^{⊤}s+\left[\begin{array}{c} x_{1,1}\\ x_{2,1} \end{array}\right]\in Z_{q}^{2m}.$$
    $$c_{2}=F_{{\mathrm{id}}_{2}}^{⊤}s+\left[\begin{array}{c} x_{1,2}\\ x_{2,2} \end{array}\right]\in Z_{q}^{2m}.$$
  The ciphertext is $c=\{c_{0},c_{1},c_{2}\}$.
(4). $\mathrm{Decrypt}(pp,sk_{{\mathrm{id}}_{1}}/sk_{{\mathrm{id}}_{2}},c$)→μ: On input the public parameter pp, a secret key ${\left(sk_{{\mathrm{id}}_{i^{\prime }}}\right)}_{i^{\prime }\in \left\{1,2\right\}}$ and the ciphertext c, do:
  - For $i^{\prime }\in \left\{1,2\right\}$, compute $\mu^{\prime }=c_{0}-e_{i^{\prime }}^{⊤}c_{i^{\prime }}$.
  - μ=1 if $|\mu^{\prime }-\left\lfloor q/2\right\rfloor |<\left\lfloor q/4\right\rfloor$. Otherwise μ=0.
  - Finally, it outputs the message μ.

### 3.2. Correctness

We firstly compute

(4) $$\begin{array}{cc} \mu^{\prime } & =c_{0}-e_{i^{\prime }}^{⊤}c_{i^{\prime }}\\ & =u^{⊤}s+x+\mu \left\lfloor q/2\right\rfloor -e_{i^{\prime }}^{⊤}\left(F_{{\mathrm{id}}_{i^{\prime }}}^{⊤}s+\left[\begin{array}{c} x_{1,i^{\prime }}\\ x_{2,i^{\prime }} \end{array}\right]\right)\\ & =\mu \left\lfloor q/2\right\rfloor +x-e_{i^{\prime }}^{⊤}\left[\begin{array}{c} x_{1,i^{\prime }}\\ x_{2,i^{\prime }} \end{array}\right] \end{array}.$$

Let $e_{i^{\prime }}=\left[\begin{array}{c} {\left(e_{i^{\prime }}\right)}_{1}\\ {\left(e_{i^{\prime }}\right)}_{2} \end{array}\right]$ where ${\left(e_{i^{\prime }}\right)}_{1},{\left(e_{i^{\prime }}\right)}_{2}\in Z^{m}$. Based on Lemma 1 and the formulas in Equations (1) and (2), the error term

(5) $$\begin{array}{cc} & |x-e_{i^{\prime }}^{⊤}\left[\begin{array}{c} x_{1,i^{\prime }}\\ x_{2,i^{\prime }} \end{array}\right]|\\ \leq & \left|x|+\right|e_{i^{\prime }}^{⊤}\left[\begin{array}{c} x_{1,i^{\prime }}\\ x_{2,i^{\prime }} \end{array}\right]|\\ = & \left|x|+\right|{\left(e_{i^{\prime }}\right)}_{1}^{⊤}x_{1,i^{\prime }}\left|+\right|{\left(e_{i^{\prime }}\right)}_{2}^{⊤}x_{2,i^{\prime }}|\\ \leq & q\alpha \omega \left(\sqrt{\log m}\right)+\frac{1}{2}+\sigma \sqrt{m}q\alpha \omega \left(\sqrt{\log m}\right)+\frac{\sigma m}{2}\\ & +\sigma \sqrt{m}\cdot O\left(B\sqrt{m}\right)\\ \leq & q\alpha \sigma m\omega \left(\sqrt{\log m}\right)+O\left(B\sigma m\right) \end{array}$$

To ensure the correctness of decryption and preform the security proof, we need that:

- the error term is less than q/5 with overwhelming probability(w.h.p)(i.e., $\alpha <{\left[\sigma m\omega \left(\sqrt{\log m}\right)\right]}^{-1}$, and q=Ω(Bσm)),
- the TrapGen algorithm can operate (i.e., m>3(1+δ)nlogq for some δ>0),
- the Leftover Hash Lemma can be applied to security proof (i.e., m>(n+1)logq+ω(logn)),
- the SampleLeft and SampleRight algorithm can operate (i.e., $\sigma >\sigma_{TG}B\sqrt{m}\omega \left(\sqrt{\log m}\right)$ where $\sigma_{TG}=O\left(\sqrt{n\log q}\right)$),
- the Regev’s LWE reduction applies (i.e., $q>2\sqrt{n}/\alpha$) and
- the security reduction applies (i.e., $\alpha qm^{5/2}\left(1+p^{p}n^{cp-c+1}\right)/B\leq \frac{p}{n+1}\cdot {\left(\frac{1}{pn^{c}}\right)}^{p+1}$ i.e., $B\geq \alpha qm^{5/2}p^{2p}n^{2cp+2}$).

### 3.3. Security

**Theorem** **2.** *If the $\left(Z_{q},n,{\bar{\Psi }}_{\alpha }\right)$-LWE assumption holds, the above IB-DRE scheme is IND-ID-CPA secure.*

**Proof.**  Let A be a probabilistic polynomial-time(PPT) adversary that can break our IB-DRE scheme with advantage ε>0. Then there exists a reduction that solves the $\left(Z_{q},n,{\bar{\Psi }}_{\alpha }\right)$-LWE problem with an negligible advantage. Let Q=Q(n) is the upper bound of the number of KeyGen queries and $I=\{\left({\mathrm{id}}_{1}^{*},{\mathrm{id}}_{2}^{*}\right),{\left({\mathrm{id}}_{1}^{յ},{\mathrm{id}}_{2}^{յ}\right)}_{յ\in [Q]}\}$ where $\left({\mathrm{id}}_{1}^{*},{\mathrm{id}}_{2}^{*}\right)$ are the challenge IDs and ${\left({\mathrm{id}}_{1}^{յ},{\mathrm{id}}_{2}^{յ}\right)}_{յ\in [Q]}$ are the queried IDs. Different from $Q\leq \frac{q}{2}$ in [11], here we let $Q\leq \frac{n^{c}}{4}-1$ where c=c(n). We show the security via the following games. In each game, we define a value r∈{0,1} and let $W_{i}$ denote the event that the adversary correctly guesses the challenge bit, i.e., the challenger outputs $r^{\prime }=r$ in ${\mathrm{Game}}_{i}$. $|\mathrm{Pr}\left[W_{i}\right]-\frac{1}{2}|$ is the adversary’s advantage.

${\mathrm{Game}}_{0}$. This is the real IND-ID-CPA game between an adversary A and the challenger. We have

$$|\mathrm{Pr}\left[W_{0}\right]-\frac{1}{2}|=|\mathrm{Pr}\left[r^{\prime }=r\right]-\frac{1}{2}|=\varepsilon .$$

${\mathrm{Game}}_{1}$. This game is as same as ${\mathrm{Game}}_{0}$ except we add an abort event at the end of the game. The challenger chooses randomly $y_{i,j},y_{i,j}^{{}^{\prime }}\leftarrow \left[1,pn^{c}\right]$ and $y_{0}\leftarrow \left[-\left(n+1\right){\left(pn^{c}\right)}^{p},0\right]$. Let $y=\{y_{0},{\left(y_{i,j}\right)}_{\left(i,j)\in [p,l\right]}\}$ and $y^{{}^{\prime }}=\left\{y_{0},{\left(y_{i,j}^{{}^{\prime }}\right)}_{\left(i,j)\in [p,l\right]}\right\}$. Let *H* and $H^{{}^{\prime }}$ be two function where

$$H\left({\mathrm{id}}_{1}\right)=y_{0}+\sum_{\left(j_{1},j_{2},\cdots ,j_{p}\right)\in F\left({\mathrm{id}}_{1}\right)}y_{i,j_{1}}\cdots y_{i,j_{p}}.$$

$$H^{{}^{\prime }}\left({\mathrm{id}}_{2}\right)=y_{0}+\sum_{\left(j_{1},j_{2},\cdots ,j_{p}\right)\in F\left({\mathrm{id}}_{2}\right)}y_{i,j_{1}}^{{}^{\prime }}\cdots y_{i,j_{p}}^{{}^{\prime }}.$$

For $I=\{\left({\mathrm{id}}_{1}^{*},{\mathrm{id}}_{2}^{*}\right),{\left({\mathrm{id}}_{1}^{յ},{\mathrm{id}}_{2}^{յ}\right)}_{j\in [Q]}\}$, the challenger checks whether the following formula holds:

$$H\left({\mathrm{id}}_{1}^{*}\right)=0\wedge H^{{}^{\prime }}\left({\mathrm{id}}_{2}^{*}\right)=0\overset{Q}{\underset{յ=1}{⋀}}\left(H\left({\mathrm{id}}_{1}^{յ}\right)\neq 0\wedge H^{{}^{\prime }}\left({\mathrm{id}}_{2}^{յ}\right)\neq 0\right).$$

If they do not hold, the game aborts, i.e., the challenger outputs a random $r^{\prime }\in \left\{0,1\right\}$. Otherwise, the challenger outputs $r^{\prime }=r$. By Lemma 8, we have

$$|\mathrm{Pr}\left[W_{1}\right]-\frac{1}{2}|\geq \frac{1}{{\left(n+1\right)}^{2}}\cdot {\left(\frac{1}{pn^{c}}\right)}^{2p}\cdot \left(\varepsilon -\frac{2Q}{n^{c}}\right).$$

**Lemma** **8** ([11,14]). Let η(I) denotes the non-abort probability, and $\eta \left(I\right)=\mathrm{Pr}[H\left({\mathrm{id}}_{1}^{*}\right)=0\wedge H^{{}^{\prime }}\left({\mathrm{id}}_{2}^{*}\right)=0{⋀}_{յ=1}^{Q}\left(H\left({\mathrm{id}}_{1}^{յ}\right)\neq 0\wedge H^{{}^{\prime }}\left({\mathrm{id}}_{2}^{յ}\right)\neq 0\right)]$. $\eta_{\min},\eta_{\max}$ denote the minimum and maximum probability of η(I), respectively. Then we have $|\mathrm{Pr}\left[W_{1}\right]-\frac{1}{2}|=\eta_{\min}\cdot \left|\mathrm{Pr}\left[W_{0}\right]-\frac{1}{2}\right|-\left(\eta_{\max}-\eta_{\min}\right)/2\geq \frac{1}{{\left(n+1\right)}^{2}}\cdot {\left(\frac{1}{pn^{c}}\right)}^{2p}\cdot \left(\varepsilon -\frac{2Q}{n^{c}}\right)$.

**Proof of Lemma 8**  For (Q+1)-tuple identities, the non-abort probability $\eta \left(I\right)=\mathrm{Pr}[H\left({\mathrm{id}}_{1}^{*}\right)=0\wedge H^{{}^{\prime }}\left({\mathrm{id}}_{2}^{*}\right)=0{⋀}_{յ=1}^{Q}\left(H\left({\mathrm{id}}_{1}^{յ}\right)\neq 0\wedge H^{{}^{\prime }}\left({\mathrm{id}}_{2}^{յ}\right)\neq 0\right)]$. As we know, the non-abort probability is taken over $y=\{y_{0},{\left(y_{i,j}\right)}_{\left(i,j)\in [p,l\right]}\}$ and $y^{{}^{\prime }}=\left\{y_{0},{\left(y_{i,j}^{{}^{\prime }}\right)}_{\left(i,j)\in [p,l\right]}\right\}$ which are chose in ${\mathrm{Game}}_{1}$. For any $y_{i,j}$ and $y_{i,j}^{{}^{\prime }}$, we can find a $y_{0}\leftarrow \left[-\left(n+1\right){\left(pn^{c}\right)}^{p},0\right]$ such that $\mathrm{Pr}[H\left({\mathrm{id}}_{1}^{*}\right)=0]$ and $\mathrm{Pr}[H^{{}^{\prime }}\left({\mathrm{id}}_{2}^{*}\right)=0]$ are

(6) $$0\leq \sum_{\left(j_{1},\cdots ,j_{p}\right)\in F\left({\mathrm{id}}_{1}^{*}\right)}y_{1,j_{1}},\cdots ,y_{p,j_{p}}\leq \sum_{\left(j_{1},\cdots ,j_{p}\right)\in F\left({\mathrm{id}}_{1}^{*}\right)}{\left(pn^{c}\right)}^{p}<\left(n+1\right){\left(pn^{c}\right)}^{p},$$

(7) $$0\leq \sum_{\left(j_{1},\cdots ,j_{p}\right)\in F\left({\mathrm{id}}_{2}^{*}\right)}y_{1,j_{1}}^{{}^{\prime }},\cdots ,y_{p,j_{p}}^{{}^{\prime }}\leq \sum_{\left(j_{1},\cdots ,j_{p}\right)\in F\left({\mathrm{id}}_{2}^{*}\right)}{\left(pn^{c}\right)}^{p}<\left(n+1\right){\left(pn^{c}\right)}^{p}.$$

We have $\mathrm{Pr}\left[H\left({\mathrm{id}}_{1}^{*}\right)=0\right]=\mathrm{Pr}\left[H^{{}^{\prime }}\left({\mathrm{id}}_{2}^{*}\right)=0\right]=\frac{1}{n+1}\cdot {\left(\frac{1}{pn^{c}}\right)}^{p}$. Therefore, the upper bound of non-abort probability η(I) is

(8) $$\eta \left(I\right)\leq \mathrm{Pr}\left[H\left({\mathrm{id}}_{1}^{*}\right)=0\wedge H^{{}^{\prime }}\left({\mathrm{id}}_{2}^{*}\right)=0\right]=\frac{1}{{\left(n+1\right)}^{2}}\cdot {\left(\frac{1}{pn^{c}}\right)}^{2p}.$$

Next we give the lower bound of non-abort probability η(I).

(9) $$\begin{array}{cc} \eta (I)= & \mathrm{Pr}[H\left({\mathrm{id}}_{1}^{*}\right)=0\wedge H^{{}^{\prime }}\left({\mathrm{id}}_{2}^{*}\right)=0\overset{Q}{\underset{յ=1}{⋀}}\left(H\left({\mathrm{id}}_{1}^{յ}\right)\neq 0\wedge H^{{}^{\prime }}\left({\mathrm{id}}_{2}^{յ}\right)\neq 0\right)]\\ = & \mathrm{Pr}\left[H\left({\mathrm{id}}_{1}^{*}\right)=0\overset{Q}{\underset{յ=1}{⋀}}H\left({\mathrm{id}}_{1}^{յ}\right)\neq 0\right]\cdot \mathrm{Pr}\left[H^{{}^{\prime }}\left({\mathrm{id}}_{2}^{*}\right)=0\overset{Q}{\underset{յ=1}{⋀}}H^{{}^{\prime }}\left({\mathrm{id}}_{2}^{յ}\right)\neq 0\right]. \end{array}$$

Then we calculate the lower bound of $\mathrm{Pr}[H\left({\mathrm{id}}_{1}^{*}\right)=0{⋀}_{յ=1}^{Q}H\left({\mathrm{id}}_{1}^{յ}\right)\neq 0]$ and $\mathrm{Pr}[H^{{}^{\prime }}\left({\mathrm{id}}_{2}^{*}\right)=0{⋀}_{յ=1}^{Q}H^{{}^{\prime }}\left({\mathrm{id}}_{2}^{յ}\right)\neq 0]$, respectively.

(10) $$\begin{array}{cc} \; & \mathrm{Pr}[H\left({\mathrm{id}}_{1}^{*}\right)=0\overset{Q}{\underset{յ=1}{⋀}}H\left({\mathrm{id}}_{1}^{յ}\right)\neq 0]\\ = & \mathrm{Pr}\left[\overset{Q}{\underset{յ=1}{⋀}}H\left({\mathrm{id}}_{1}^{յ}\right)\neq 0|H\left({\mathrm{id}}_{1}^{*}\right)=0\right]\cdot \mathrm{Pr}\left[H\left({\mathrm{id}}_{1}^{*}\right)=0\right]\\ = & \left(1-\mathrm{Pr}[\overset{Q}{\underset{յ=1}{⋁}}H\left({\mathrm{id}}_{1}^{յ}\right)=0|H\left({\mathrm{id}}_{1}^{*}\right)=0]\right)\cdot \mathrm{Pr}\left[H\left({\mathrm{id}}_{1}^{*}\right)=0\right]\\ \geq & \left(1-\sum_{յ\in Q}\mathrm{Pr}\left[H\left({\mathrm{id}}_{1}^{յ}\right)=0|H\left({\mathrm{id}}_{1}^{*}\right)=0\right]\right)\cdot \mathrm{Pr}\left[H\left({\mathrm{id}}_{1}^{*}\right)=0\right]\\ = & \frac{1}{n+1}\cdot {\left(\frac{1}{pn^{c}}\right)}^{p}\cdot \left(1-\sum_{յ\in Q}\mathrm{Pr}\left[H\left({\mathrm{id}}_{1}^{յ}\right)=0|H\left({\mathrm{id}}_{1}^{*}\right)=0\right]\right)\\ \geq & \frac{1}{n+1}\cdot {\left(\frac{1}{pn^{c}}\right)}^{p}\cdot \left(1-\frac{Q}{n^{c}}\right). \end{array}$$

The last equation follows the fact that since $H({\mathrm{id}}_{1}^{*})=0$, so we have $y_{0}=-\sum_{\left(j_{1},\cdots ,j_{p}\right)\in F\left({\mathrm{id}}_{1}^{*}\right)}y_{1,j_{1}},\cdots ,y_{p,j_{p}}$. Then the probability of

(11) $$\begin{array}{cc} \; & \mathrm{Pr}\left[H\left({\mathrm{id}}_{1}^{յ}\right)=0|H\left({\mathrm{id}}_{1}^{*}\right)=0\right]=\mathrm{Pr}\left[y_{0}+\sum_{\left(j_{1},\cdots ,j_{p}\right)\in F\left({\mathrm{id}}_{յ}\right)}y_{1,j_{1}},\cdots ,y_{p,j_{p}}=0\right]\\ = & \mathrm{Pr}\left[-\sum_{\left(j_{1},\cdots ,j_{p}\right)\in F\left({\mathrm{id}}_{1}^{*}\right)}y_{1,j_{1}},\cdots ,y_{p,j_{p}}+\sum_{\left(j_{1},\cdots ,j_{p}\right)\in F\left({\mathrm{id}}_{1}^{յ}\right)}y_{1,j_{1}},\cdots ,y_{p,j_{p}}=0\right]. \end{array}$$

Since ${\mathrm{id}}_{1}^{*}\neq {\mathrm{id}}_{1}^{յ}$ and F is an injective function, then $F\left({\mathrm{id}}_{1}^{յ}\right)\neq F\left({\mathrm{id}}_{1}^{*}\right)$, the equation $\sum_{\left(j_{1},\cdots ,j_{p}\right)\in F\left({\mathrm{id}}_{1}^{յ}\right)}y_{1,j_{1}},\cdots ,y_{p,j_{p}}-\sum_{\left(j_{1},\cdots ,j_{p}\right)\in F\left({\mathrm{id}}_{1}^{*}\right)}y_{1,j_{1}},\cdots ,y_{p,j_{p}}$ can be seen as a polynomial with degree *p*. Since $y_{i,j}\leftarrow \left[1,pn^{c}\right]$, the probability of

(12) $$\mathrm{Pr}\left[-\sum_{\left(j_{1},\cdots ,j_{p}\right)\in F\left({\mathrm{id}}_{1}^{*}\right)}y_{1,j_{1}},\cdots ,y_{p,j_{p}}+\sum_{\left(j_{1},\cdots ,j_{p}\right)\in F\left({\mathrm{id}}_{1}^{յ}\right)}y_{1,j_{1}},\cdots ,y_{p,j_{p}}=0\right]\leq \frac{p}{pn^{c}}=\frac{1}{n^{c}}.$$

By the same method, we can get $\mathrm{Pr}\left[H^{{}^{\prime }}\left({\mathrm{id}}_{2}^{*}\right)=0{⋀}_{յ=1}^{Q}H^{{}^{\prime }}\left({\mathrm{id}}_{2}^{յ}\right)\neq 0\right]=\frac{1}{n+1}\cdot {\left(\frac{1}{pn^{c}}\right)}^{p}\cdot \left(1-\frac{Q}{n^{c}}\right)$. Finally, the non-abort probability

(13) $$\eta \left(I\right)=\mathrm{Pr}\left[H\left({\mathrm{id}}_{1}^{*}\right)=0\overset{Q}{\underset{յ=1}{⋀}}H\left({\mathrm{id}}_{1}^{յ}\right)\neq 0\right]\cdot \mathrm{Pr}\left[H^{{}^{\prime }}\left({\mathrm{id}}_{2}^{*}\right)=0\overset{Q}{\underset{յ=1}{⋀}}H^{{}^{\prime }}\left({\mathrm{id}}_{2}^{յ}\right)\neq 0\right]\geq \frac{1}{{\left(n+1\right)}^{2}}\cdot {\left(\frac{1}{pn^{c}}\right)}^{2p}\cdot {\left(1-\frac{Q}{n^{c}}\right)}^{2}.$$

Then we have $\eta_{\min}=\frac{1}{{\left(n+1\right)}^{2}}\cdot {\left(\frac{1}{pn^{c}}\right)}^{2p}\cdot {\left(1-\frac{Q}{n^{c}}\right)}^{2}$, $\eta_{\max}=\frac{1}{{\left(n+1\right)}^{2}}\cdot {\left(\frac{1}{pn^{c}}\right)}^{2p}$. Finally,

(14) $$\begin{array}{cc} \; & |\mathrm{Pr}\left[W_{1}\right]-\frac{1}{2}|\geq \eta_{\min}\cdot \varepsilon -\left(\eta_{\max}-\eta_{\min}\right)/2\\ = & \frac{1}{{\left(n+1\right)}^{2}}\cdot {\left(\frac{1}{pn^{c}}\right)}^{2p}\cdot {\left(1-\frac{Q}{n^{c}}\right)}^{2}\cdot \varepsilon -\frac{\frac{1}{{\left(n+1\right)}^{2}}\cdot {\left(\frac{1}{pn^{c}}\right)}^{2p}-\frac{1}{{\left(n+1\right)}^{2}}\cdot {\left(\frac{1}{pn^{c}}\right)}^{2p}\cdot {\left(1-\frac{Q}{n^{c}}\right)}^{2}}{2}\\ = & \frac{1}{{\left(n+1\right)}^{2}}\cdot {\left(\frac{1}{pn^{c}}\right)}^{2p}\cdot {\left(1-\frac{Q}{n^{c}}\right)}^{2}\cdot \varepsilon -\frac{1}{{\left(n+1\right)}^{2}}\cdot {\left(\frac{1}{pn^{c}}\right)}^{2p}\left(\frac{1}{2}-\frac{1}{2}{\left(1-\frac{Q}{n^{c}}\right)}^{2}\right)\\ \geq & \frac{1}{{\left(n+1\right)}^{2}}\cdot {\left(\frac{1}{pn^{c}}\right)}^{2p}\cdot \left(\varepsilon -\frac{2Q}{n^{c}}\right) \end{array}$$

The last inequality due to that $\varepsilon \leq \frac{1}{2}$. ☐

${\mathrm{Game}}_{2}$. In this game, we change the way $A_{i,j},A_{i,j}^{{}^{\prime }}$ and $B_{0}$ are generated. The challenger firstly chooses $y=\{y_{0},{\left(y_{i,j}\right)}_{\left(i,j)\in [p,l\right]}\}$, $y^{{}^{\prime }}=\left\{y_{0},{\left(y_{i,j}^{{}^{\prime }}\right)}_{\left(i,j)\in [p,l\right]}\right\}$ as ${\mathrm{Game}}_{1}$ and then chooses three matrices $R_{0},R_{i,j},R_{i,j}^{{}^{\prime }}\in {\left\{-1,1\right\}}^{m\times m}$. Compute

(15) $$B_{0}=A_{0}R_{0}+y_{0}G$$

(16) $$A_{i,j}=A_{0}R_{i,j}+y_{i,j}G$$

(17) $$A_{i,j}^{{}^{\prime }}=A_{0}R_{i,j}^{{}^{\prime }}+y_{i,j}^{{}^{\prime }}G$$

Based on Lemma 6, the distribution (A, $A_{0}R_{0}+y_{0}G$, $A_{0}R_{i,j}+y_{i,j}G$, $A_{0}R_{i,j}^{{}^{\prime }}+y_{i,j}^{{}^{\prime }}G$) is negl(n)-close to the distribution (A, $B_{0}$, $A_{i,j}$, $A_{i,j}^{{}^{\prime }}$). Therefore, we have

$$|\mathrm{Pr}\left[W_{1}\right]-\mathrm{Pr}\left[W_{2}\right]|=\mathrm{negl}\left(n\right).$$

Before the next game, for any id∈ID, we make a definition as follow. Let

$$R_{{\mathrm{id}}_{1}}=R_{0}+\sum_{\left(j_{1},\cdots ,j_{p}\right)\in F\left({\mathrm{id}}_{1}\right)}{\mathrm{TrapEval}}_{p}\left(R_{1,j_{1}},\cdots ,R_{p,j_{p}}\right),$$

$$R_{{\mathrm{id}}_{2}}=R_{0}+\sum_{\left(j_{1},\cdots ,j_{p}\right)\in F\left({\mathrm{id}}_{2}\right)}{\mathrm{TrapEval}}_{p}\left(R_{1,j_{1}}^{{}^{\prime }},\cdots ,R_{p,j_{p}}^{{}^{\prime }}\right).$$

Based on Lemma 3, we have

(18) $$\begin{array}{cc} \; & ∥R_{{\mathrm{id}}_{1}}∥\leq ∥R_{0}∥+\sum_{\left(j_{1},\cdots ,j_{p}\right)\in F\left({\mathrm{id}}_{1}\right)}∥{\mathrm{TrapEval}}_{p}\left(R_{1,j_{1}},\cdots ,R_{p,j_{p}}\right)∥\\ \leq & m+nmp{\left(pn^{c}\right)}^{p-1}=m\left(1+p^{p}n^{cp-c+1}\right). \end{array}$$

$∥R_{{\mathrm{id}}_{2}}∥$ is the same as $∥R_{{\mathrm{id}}_{1}}∥$.

${\mathrm{Game}}_{3}$. In this game, we show that when we change the ciphertext is generated, the distributions $x_{2,i^{\prime }}$ and $R_{{\mathrm{id}}_{i^{\prime }}^{*}}^{⊤}x_{1,i^{\prime }}+x_{2,i^{\prime }}$ are within statistical distance $\alpha qm^{5/2}\left(1+p^{p}n^{cp-c+1}\right)/B$ where $i^{\prime }\in \left\{1,2\right\}$. The challenge ciphertext is generated as follows: the challenger firstly chooses $s\in Z_{q}^{n}$, $x\overset{{\bar{\Psi }}_{\alpha }}{\leftarrow }Z_{q}^{n}$, $x_{1,1},x_{1,2},\overset{{\bar{\Psi }}_{\alpha }^{m}}{\leftarrow }Z_{q}^{m}$, $x_{2,1},x_{2,2}\leftarrow {\left\{-B,B\right\}}^{m}$ and computes $R_{{\mathrm{id}}_{1}^{*}}$, $R_{{\mathrm{id}}_{2}^{*}}$. The challenge ciphertext

$$c_{0}^{*}=u^{⊤}s+x+\mu^{*}\left\lfloor q/2\right\rfloor ,$$

$$c_{1}^{*}={\left(A_{0}|H\left({\mathrm{id}}_{1}^{*}\right)\right)}^{⊤}s+\left[\begin{array}{c} x_{1,1}\\ R_{{\mathrm{id}}_{1}^{*}}^{⊤}x_{1,1}+x_{2,1} \end{array}\right],$$

$$c_{2}^{*}={\left(A_{0}|H\left({\mathrm{id}}_{2}^{*}\right)\right)}^{⊤}s+\left[\begin{array}{c} x_{1,2}\\ R_{{\mathrm{id}}_{2}^{*}}^{⊤}x_{1,2}+x_{2,2} \end{array}\right].$$

$\mu^{*}\in \left\{0,1\right\}$ is the message chosen by A.

Since $x_{1,i^{\prime }}\overset{{\bar{\Psi }}_{\alpha }^{m}}{\leftarrow }$, by the formula in Equation (18) we have

(19) $$\begin{array}{cc} ∥R_{{\mathrm{id}}_{i^{\prime }}^{*}}^{⊤}x_{1,i^{\prime }}{∥}_{\infty }\leq & ∥R_{{\mathrm{id}}_{i^{\prime }}^{*}}^{⊤}x_{1,i^{\prime }}∥\leq ∥R_{{\mathrm{id}}_{i^{\prime }}^{*}}^{⊤}∥\cdot ∥x_{1,i^{\prime }}∥\\ \leq & m\left(1+p^{p}n^{cp-c+1}\right)\cdot \alpha q\sqrt{m}\\ = & \alpha qm^{3/2}\left(1+p^{p}n^{cp-c+1}\right) \end{array}$$

By Lemma 7, let $x_{2,i^{\prime }}$ be a fixed vector, the distributions $x_{2,i^{\prime }}$ and $R_{{\mathrm{id}}_{i^{\prime }}^{*}}^{⊤}x_{1,i^{\prime }}+x_{2,i^{\prime }}$ are within statistical distance $\alpha qm^{5/2}\left(1+p^{p}n^{cp-c+1}\right)/B\leq \frac{p}{n+1}\cdot {\left(\frac{1}{pn^{c}}\right)}^{p+1}$.

${\mathrm{Game}}_{4}$. In this game, we change the way $A_{0}$ is generated. The challenger chooses a random matrix $A_{0}\in Z_{q}^{n\times m}$ instead of using the TrapGen algorithm. For the secret key queries, the challenger respond by the SampleRight instead of SampleLeft. By the definition of $R_{\mathrm{id}}$, we have $H\left({\mathrm{id}}_{1}\right)=A_{0}\cdot \left(R_{{\mathrm{id}}_{1}}+H\left({\mathrm{id}}_{1}\right)G\right)$, $H^{{}^{\prime }}\left({\mathrm{id}}_{2}\right)=A_{0}\cdot \left(R_{{\mathrm{id}}_{2}}+H^{{}^{\prime }}\left({\mathrm{id}}_{2}\right)G\right)$. If $H({\mathrm{id}}_{1})=0$ or $H^{{}^{\prime }}\left({\mathrm{id}}_{2}\right)=0$, the challenger aborts and returns a random bit. Otherwise, it returns $e_{1}$ and $e_{2}$ to A where

$$e_{1}\leftarrow \mathrm{SampleRight}\left(A_{0},G,R_{{\mathrm{id}}_{1}},H\left({\mathrm{id}}_{1}\right),u,T_{G}\right)$$

$$e_{2}\leftarrow \mathrm{SampleRight}\left(A_{0},G,R_{{\mathrm{id}}_{2}},H^{{}^{\prime }}\left({\mathrm{id}}_{2}\right),u,T_{G}\right).$$

In particular, the challenger checks if the challenge identity $\left({\mathrm{id}}_{1}^{*},{\mathrm{id}}_{2}^{*}\right)$ satisfies $H({\mathrm{id}}_{1}^{*})=0$ and $H^{{}^{\prime }}\left({\mathrm{id}}_{2}^{*}\right)=0$. If not, the game aborts as in ${\mathrm{Game}}_{1}$.

Since in the adversary’s view, ${\mathrm{Game}}_{2}$ and ${\mathrm{Game}}_{4}$ are identical (the public parameters, abort conditions, responses to private key queries and the challenge ciphertext). The advantage of the adversary A is identical to ${\mathrm{Game}}_{2}$, i.e.,

$$|\mathrm{Pr}\left[W_{2}\right]-\mathrm{Pr}\left[W_{4}\right]|=\mathrm{negl}\left(n\right).$$

${\mathrm{Game}}_{5}$. As we know, the ciphertext space is $C\in Z_{q}\times Z_{q}^{2m}\times Z_{q}^{2m}$. In this game, the challenger set the ciphertext as $c^{*}=\left\{c_{0},c_{1}^{*},c_{2}^{*}\right\}$ which is uniformly random in $Z_{q}\times Z_{q}^{2m}\times Z_{q}^{2m}$ in the challenge phase. The advantage of the adversary A is 0. As shown in Lemma 9, assuming $\left(Z_{q},n,{\bar{\Psi }}_{\alpha }\right)$-LWE holds, ${\mathrm{Game}}_{4}$ and ${\mathrm{Game}}_{5}$ are computationally indistinguishable, i.e., $|\mathrm{Pr}\left[W_{4}\right]-\left|\mathrm{Pr}\left[W_{5}\right]\right|=\mathrm{negl}\left(n\right).$ ☐

**Lemma** **9.** *For any PPT adversary A, there exists a challenger B such that*

$$|\mathrm{Pr}\left[W_{4}\right]-\mathrm{Pr}\left[W_{5}\right]\leq \mathrm{LWE}-Adv\left(B\right).$$

**Proof of Lemma** **9.** Suppose A has a non-negligible advantage in distinguishing ${\mathrm{Game}}_{4}$ and ${\mathrm{Game}}_{5}$. We use A to construct an LWE algorithm denoted B.

Recall from Definition 4 that an LWE problem instance is provided as a sampling oracle O which is either a truly random sampler $O_{s}^{{}^{\prime }}$ or a noisy pseudo-random sampler $O_{s}$ for a secret $s\in Z_{q}^{n}$. The challenger B uses the adversary A to distinguish which the sampler it is given, and proceeds as follows:

**Instance**. The challenger B requests from O to obtains (m+1) LWE samples that we denote as:

$$\left\{\left(w_{0},v_{0}\right),\left(w_{1},v_{1}\right),\left(w_{2},v_{2}\right),\ldots ,\left(w_{m},v_{m}\right)\right\}\in \left(Z_{q}^{n}\times Z_{q}\right)$$

**Setup**. The challenger B constructs the public parameters pp as follows:

(1) Construct a matrix $A_{0}\in Z_{q}^{n\times m}$ by assembling *m* LWE samples such that $A_{0}=\left(w_{1},w_{2},\ldots ,w_{m}\right)$, and let $u=w_{0}$.
(2) Choose y as in ${\mathrm{Game}}_{1}$ and constructs the remainder of the public parameters as in ${\mathrm{Game}}_{2}$.
(3) Send the $pp=\{A_{0},{\left(A_{i,j}\right)}_{\left(i,j)\in [p,l\right]},{\left(A_{i,j}^{{}^{\prime }}\right)}_{\left(i,j)\in [p,l\right]},B_{0},u\}$ to A.

**Queries**. A makes secret key query. The challenger B computes and checks if H(id)=0. If it holds, it aborts. Otherwise it generate the secret key for A as in ${\mathrm{Game}}_{4}$.

**Challenge**. A sends a message bit $\mu^{*}\in \left\{0,1\right\}$ and the target identities $\left({\mathrm{id}}_{1}^{*},{\mathrm{id}}_{2}^{*}\right)$ to B. The challenger B constructs $v^{*}={\left(v_{1},v_{2},\cdots ,v_{m}\right)}^{⊤}$ where $v_{0},v_{1},\cdots ,v_{m}\in Z_{q}$ is the LWE samples. B chooses $x_{2,1},x_{2,2}\leftarrow {\left\{-B,B\right\}}^{m}$. The challenge ciphertext

$$c_{0}^{*}=v_{0}+\mu^{*}\left\lfloor q/2\right\rfloor$$

$$c_{1}^{*}=\left[\begin{array}{c} v^{*}+0_{m}\\ R_{{\mathrm{id}}_{1}^{*}}^{⊤}v^{*}+x_{2,1} \end{array}\right]$$

$$c_{2}^{*}=\left[\begin{array}{c} v^{*}+0_{m}\\ R_{{\mathrm{id}}_{2}^{*}}^{⊤}v^{*}+x_{2,2} \end{array}\right].$$

B sends the challenge ciphertext $c^{*}=\left\{c_{0}^{*},c_{1}^{*},c_{2}^{*}\right\}$ to A.

Note that when $O=O_{s}$, the ciphertext is valid.(We just argue only when no abort happens). Since $H({\mathrm{id}}_{1}^{*})=0$ and $H^{{}^{\prime }}\left({\mathrm{id}}_{2}^{*}\right)=0$, we have $F_{{\mathrm{id}}_{1}^{*}}=\left[A_{0}|H\left({\mathrm{id}}_{1}^{*}\right)\right]=\left[A_{0}|A_{0}R_{{\mathrm{id}}_{1}^{*}}\right]$ where $H\left({\mathrm{id}}_{1}^{*}\right)=A_{0}\cdot \left(R_{{\mathrm{id}}_{1}^{*}}+H\left({\mathrm{id}}_{1}^{*}\right)G\right)=A_{0}\cdot R_{{\mathrm{id}}_{1}^{*}}$. The same as $F_{{\mathrm{id}}_{1}^{*}}$, we have $F_{{\mathrm{id}}_{2}^{*}}=\left[A_{0}|A_{0}R_{{\mathrm{id}}_{2}^{*}}\right]$. By definition of $O_{s}$, we know $v^{*}=A_{0}^{⊤}s+x$. Then we have

(20) $$c_{0}^{*}=v_{0}+\mu^{*}\left\lfloor q/2\right\rfloor =\left(u^{⊤}s+x_{0}\right)+\mu^{*}\left\lfloor q/2\right\rfloor$$

(21) $$\begin{array}{c} c_{1}^{*}=\left[\begin{array}{c} v^{*}+0_{m}\\ R_{{\mathrm{id}}_{1}^{*}}^{⊤}v^{*}+x_{2,1} \end{array}\right]=\left[\begin{array}{c} A_{0}^{⊤}s+x+0_{m}\\ R_{{\mathrm{id}}_{1}^{*}}^{⊤}\left(A_{0}^{⊤}s+x\right)+x_{2,1} \end{array}\right]=\left[\begin{array}{c} A_{0}^{⊤}s+x+0_{m}\\ {\left(A_{0}R_{{\mathrm{id}}_{1}^{*}}\right)}^{⊤}s+R_{{\mathrm{id}}_{1}^{*}}^{⊤}x+x_{2,1} \end{array}\right]=F_{{\mathrm{id}}_{1}^{*}}^{⊤}s+\left[\begin{array}{c} x\\ R_{{\mathrm{id}}_{1}^{*}}^{⊤}x+x_{2,1} \end{array}\right] \end{array}$$

The same as $c_{1}^{*}$, $c_{2}^{*}$ is also valid. It is also similar to the ${\mathrm{Game}}_{3}$.

When $O=O_{s}^{{}^{\prime }}$, $v_{0}\in Z_{q}$ and $v^{*}\in Z_{q}^{m}$ are all uniform. Therefore $c_{0}^{*},c_{1}^{*},c_{2}^{*}$ are uniform in $\in Z_{q}\times Z_{q}^{2m}\times Z_{q}^{2m}$ by the Lemma 6.

**Guess**. After being allowed to make additional secret key queries, A guesses if it is interacting with a ${\mathrm{Game}}_{4}$ or ${\mathrm{Game}}_{5}$ challenger. B output A’s guess as the answer to the LWE challenge it is trying to solve. Therefore we have $\mathrm{LWE}-Adv\left(B\right)=|\mathrm{Pr}\left[W_{4}\right]-\mathrm{Pr}\left[W_{5}\right]|$. ☐

## 4. Adaptively Secure Hierarchical IB-DRE Scheme with Short Public Parameter

To lighten the pressure of the KGC, hierarchical IBE (HIBE) scheme was proposed. In HIBE, the user’s identity can be described by an identity tuple, and we let ${\mathrm{ID}}_{k}=\left({\mathrm{id}}_{1},{\mathrm{id}}_{2},\cdots ,{\mathrm{id}}_{k}\right)$ denote an identity at the depth *k*. There are many users at each depth. In this section, we use ${\mathrm{ID}}_{k,1}$ and ${\mathrm{ID}}_{k,2}$ to denote the two arbitrary receivers at the depth *k* in our HIB-DRE scheme.

As we all know, when convert an selectively secure HIBE scheme in [11] to an adaptively secure HIBE, for an identity ${\mathrm{ID}}_{k}=\left({\mathrm{id}}_{1},{\mathrm{id}}_{2},\cdots ,{\mathrm{id}}_{k}\right)$ at the depth *k*, the key generation matrix/encryption matrix would be

$$F_{{\mathrm{ID}}_{k}}=[A_{0}|B_{0}+\sum_{j=1}^{n}b_{1,j}A_{1,j}\left|\cdots \right|B_{0}+\sum_{j=1}^{n}b_{k,j}A_{k,j}]\in Z_{q}^{n\times (k+1)m}.$$

where ${\left(A_{i,j}\right)}_{i\in [k],j\in [n]}$ is the matrices in the public parameters.

Therefore the public parameters would be 2dn+2 matrices which lead to a high storage cost. *d* is the maximum hierarchy depth.

In this section, we construct an adaptively secure HIB-DRE scheme with short public parameters. There are also four algorithms in this scheme: Setup, KeyGen, Encrypt and Decrypt. In this scheme, we use the same injective map function and homomorphic computation technique to reduce the size of public parameters from 2dn+2 matrices to $2dpn^{\frac{1}{p}}+2$ matrices. Different to the adaptively secure IB-DRE scheme in Section 3, we use the SampleBasisLeft [11] algorithm to generate the user’s secret key, and SampleBasisRight algorithm for the security proof. In the KeyGen phase, it needs to input a secret key for the identity at depth l−1, and then outputs a secret key for the identity at depth *l*.

$\mathrm{SampleBasisLeft}(M_{1},M,T_{M_{1}},\sigma$): On input two matrices $M_{1}\in Z_{q}^{n\times m_{1}}$, $M\in Z_{q}^{n\times m}$, a “short” basis $T_{M_{1}}$ of $\Lambda_{q}^{⊥}\left(M_{1}\right)$ and a Gaussian parameter $\sigma \geq ∥\tilde{T_{M_{1}}}∥\omega \left(\sqrt{\log m+m_{1}}\right)$. This algorithm outputs a short basis E of $\Lambda_{q}^{⊥}\left(F_{1}\right)$ where $F_{1}=\left[M_{1}|M\right]$.

$\mathrm{SampleBasisRight}(A_{0},G_{\mathrm{id}},R,T_{G},\sigma_{k}$): On input three matrices $A_{0}\in Z_{q}^{n\times m}$, $G_{\mathrm{id}}\in Z_{q}^{n\times m_{1}}$, $R\in Z_{q}^{m\times m_{1}}$, a basis $T_{G}$ of $\Lambda_{q}^{⊥}\left(G\right)$ and a Gaussian parameter $\sigma_{k}>∥\tilde{T_{G}}∥\cdot s_{R}\omega \left(\sqrt{\log m}\right)$ where $G_{\mathrm{id}}=H\left(\mathrm{id}\right)G$ and $s_{R}=∥\tilde{R}∥$. This algorithm outputs a short basis E of $\Lambda_{q}^{⊥}\left(F_{2}\right)$ where $F_{2}=\left[A_{0}|A_{0}R+G_{\mathrm{id}}\right]$.

### 4.1. Our Construction

The adaptively secure HIB-DRE scheme with short public parameters consists the following four algorithms.

(1). $\mathrm{Setup}(d,1^{n}$)→ (pp,msk): on input the maximum hierarchy depth *d* and the security parameter $1^{n}$. This algorithm outputs the public parameters pp and master secret key msk, do:
  - Perform algorithm TrapGen to generate a uniformly matrix $A_{0}\in Z_{q}^{n\times m}$ and a trapdoor $T_{A_{0}}\in Z^{m\times m}$.
  - For all identities ${\left({\mathrm{ID}}_{k}\right)}_{k\in [d]}={\left({\mathrm{id}}_{1},{\mathrm{id}}_{2},\cdots ,{\mathrm{id}}_{k}\right)}_{k\in [d]}$ where ${\mathrm{id}}_{k}\in {\left\{0,1\right\}}^{n}$, select an injective map F that maps an identity to a subset $F({\mathrm{ID}}_{k})$ of ${\left[1,l\right]}^{p}$ where $l=\lceil n^{\frac{1}{p}}\rceil$.
  - For (i,j)∈[p,l] and k∈[d], select 2dpl matrices $A_{i,j}^{k}$, ${\bar{A}}_{i,j}^{k}$.
  - Select a uniformly random vector $u={\left(u_{1},u_{2},\ldots ,u_{n}\right)}^{⊤}\in Z_{q}^{n}$ and a uniformly random matrix $B_{0}\in Z_{q}^{n\times m}$.
  The public parameter pp = $\{A_{0}$, ${\left(A_{i,j}^{k},{\bar{A}}_{i,j}^{k}\right)}_{\left(i,j)\in [p,l],k\in [d\right]}$, $B_{0},u\}$, the master secret key $msk=\{T_{A_{0}}\}$.
  For two arbitrary receivers ${\mathrm{ID}}_{k,1}$, ${\mathrm{ID}}_{k,2}$ at the depth *k*. Recall that by the function ${\mathrm{Eval}}_{p}$: ${\left(Z_{q}^{n\times m}\right)}^{p}\to Z_{q}^{n\times m}$, for the two identities $F\left({\mathrm{ID}}_{k,1}\right),F\left({\mathrm{ID}}_{k,2}\right)$, let
  $$B_{i,j}^{k}=\sum_{\left(j_{1},j_{2},\cdots ,j_{p}\right)\in F\left({\mathrm{ID}}_{k,1}\right)}{\mathrm{Eval}}_{p}\left(A_{1,j_{1}}^{k},A_{2,j_{2}}^{k},\cdots ,A_{p,j_{p}}^{k}\right),$$
  $${\bar{B}}_{i,j}^{k}=\sum_{\left(j_{1},j_{2},\cdots ,j_{p}\right)\in F\left({\mathrm{ID}}_{k,2}\right)}{\mathrm{Eval}}_{p}\left({\bar{A}}_{1,j_{1}}^{k},{\bar{A}}_{2,j_{2}}^{k},\cdots ,{\bar{A}}_{p,j_{p}}^{k}\right).$$
  Then construct
  $$H\left({\mathrm{ID}}_{k,1}\right)=B_{0}+B_{i,j}^{1}|B_{0}+B_{i,j}^{2}\left|\cdots \right|B_{0}+B_{i,j}^{k},$$
  $$H\left({\mathrm{ID}}_{k,2}\right)=B_{0}+{\bar{B}}_{i,j}^{1}|B_{0}+{\bar{B}}_{i,j}^{2}\left|\cdots \right|B_{0}+{\bar{B}}_{i,j}^{k}.$$
  Note that for $i^{\prime }\in \left\{1,2\right\}$, $F_{{\mathrm{ID}}_{k,i^{\prime }}}=\left[A_{0}|H\left({\mathrm{ID}}_{k,i^{\prime }}\right)\right]\in Z_{q}^{n\times (k+1)m}$.
(2). $\mathrm{KeyGen}(pp,{\mathrm{ID}}_{k,i^{\prime }},sk_{{\mathrm{ID}}_{k-1,i^{\prime }}},msk$)$\to sk_{{\mathrm{ID}}_{k,i^{\prime }}}$: On input the public parameter pp, the user’s identity ${\mathrm{ID}}_{k,i^{\prime }}$ at depth *k* where $i^{\prime }\in \left\{1,2\right\}$, the secret key $sk_{{\mathrm{ID}}_{k-1,i^{\prime }}}$ corresponding to an identity ${\mathrm{ID}}_{k-1,i^{\prime }}$ at depth k−1 and the master secret key msk. This algorithm KeyGen outputs a secret key $sk_{{\mathrm{ID}}_{k,i^{\prime }}}$ as follow:
  $$E_{1}\leftarrow \mathrm{SampleBasisLeft}\left(F_{{\mathrm{ID}}_{k-1,1}},B_{0}+B_{i,j}^{k},sk_{{\mathrm{ID}}_{k-1,1}},\sigma_{k}\right).$$
  $$E_{2}\leftarrow \mathrm{SampleBasisLeft}\left(F_{{\mathrm{ID}}_{k-1,2}},B_{0}+{\bar{B}}_{i,j}^{k},sk_{{\mathrm{ID}}_{k-1,2}},\sigma_{k}\right).$$
  The secret key is $sk_{{\mathrm{ID}}_{k,i^{\prime }}}=E_{i^{\prime }}$.
(3). $\mathrm{Encrypt}(pp,{\mathrm{ID}}_{k,1},{\mathrm{ID}}_{k,2},\mu$)→c: On input the public parameter pp, the user’s identities ${\mathrm{ID}}_{k,1},{\mathrm{ID}}_{k,2}$ and the message bit μ∈{0,1}. This algorithm outputs the ciphertext c. It works as follows:
  - It firstly gets $F_{{\mathrm{ID}}_{k,1}}=\left[A_{0}|H\left({\mathrm{ID}}_{k,1}\right)\right]$ and $F_{{\mathrm{ID}}_{k,2}}=\left[A_{0}|H\left({\mathrm{ID}}_{k,2}\right)\right]$ as above.
  - Choose a randomly uniform vector $s\in Z_{q}^{n}$, and a uniformly random matrix $R\leftarrow {\left\{-1,1\right\}}^{m\times km}$.
  - Choose error terms $x\overset{{\bar{\Psi }}_{\alpha_{k}}}{\leftarrow }Z_{q}$, $x_{1,1},x_{1,2},\overset{{\bar{\Psi }}_{\alpha_{k}}^{m}}{\leftarrow }Z_{q}^{m}$. Let $x_{2,1}=R^{⊤}x_{1,1},x_{2,2}=R^{⊤}x_{1,2}$, compute
    $$c_{0}=u^{⊤}s+x+\mu \left\lfloor q/2\right\rfloor \in Z_{q}.$$
    $$c_{1}=F_{{\mathrm{ID}}_{k,1}}^{⊤}s+\left[\begin{array}{c} x_{1,1}\\ x_{2,1} \end{array}\right]\in Z_{q}^{(k+1)m}.$$
    $$c_{2}=F_{{\mathrm{ID}}_{k,2}}^{⊤}s+\left[\begin{array}{c} x_{1,2}\\ x_{2,2} \end{array}\right]\in Z_{q}^{(k+1)m}.$$
  The ciphertext is $c=\{c_{0},c_{1},c_{2}\}$.
(4). $\mathrm{Decrypt}(pp,sk_{{\mathrm{ID}}_{k,i^{\prime }}},c$)→μ: On input the public parameter pp, a secret key ${\left(sk_{{\mathrm{ID}}_{k,i^{\prime }}}\right)}_{i^{\prime }\in \left\{1,2\right\}}$ where ${\mathrm{ID}}_{k,i^{\prime }}$ at depth *k* and the ciphertext c, do:
  - Set ${\bar{\sigma }}_{k}=\sigma_{k}\sqrt{(k+1)m}\omega \left(\sqrt{\log km}\right)$.
  - For $i^{\prime }\in \left\{1,2\right\}$, set $e_{{\mathrm{ID}}_{k,i^{\prime }}}\leftarrow \mathrm{SamplePre}\left(F_{{\mathrm{ID}}_{k,i^{\prime }}},sk_{{\mathrm{ID}}_{k,i^{\prime }}},u,{\bar{\sigma }}_{k}\right)$. Then $F_{{\mathrm{ID}}_{k,i^{\prime }}}e_{{\mathrm{ID}}_{k,i^{\prime }}}=u$ and $∥e_{{\mathrm{ID}}_{k,i^{\prime }}}\leq {\bar{\sigma }}_{k}\sqrt{(k+1)m}∥$.
  - Compute $\mu^{\prime }=c_{0}-e_{{\mathrm{ID}}_{k,i^{\prime }}}^{⊤}c_{i^{\prime }}\in Z_{q}$.
  - μ=1 if $|\mu^{\prime }-\left\lfloor q/2\right\rfloor |<\left\lfloor q/4\right\rfloor$. Otherwise μ=0.
  - Finally, it outputs the message μ.

### 4.2. Correctness

We firstly compute

(22) $$\begin{array}{cc} \mu^{\prime } & =c_{0}-e_{{\mathrm{ID}}_{k,i^{\prime }}}^{⊤}c_{i^{\prime }}\\ & =u^{⊤}s+x+\mu \left\lfloor q/2\right\rfloor -e_{{\mathrm{ID}}_{k,i^{\prime }}}^{⊤}\left(F_{{\mathrm{ID}}_{k,i^{\prime }}}^{⊤}s+\left[\begin{array}{c} x_{1,i^{\prime }}\\ x_{2,i^{\prime }} \end{array}\right]\right)\\ & =\mu \left\lfloor q/2\right\rfloor +x-e_{{\mathrm{ID}}_{k,i^{\prime }}}^{⊤}\left[\begin{array}{c} x_{1,i^{\prime }}\\ x_{2,i^{\prime }} \end{array}\right] \end{array}$$

Let $e_{{\mathrm{ID}}_{k,i^{\prime }}}=\left[\begin{array}{c} {\left(e_{{\mathrm{ID}}_{k,i^{\prime }}}\right)}_{1}\\ {\left(e_{{\mathrm{ID}}_{k,i^{\prime }}}\right)}_{2} \end{array}\right]$ where ${\left(e_{{\mathrm{ID}}_{k,i^{\prime }}}\right)}_{1}\in Z^{m},{\left(e_{{\mathrm{ID}}_{k,i^{\prime }}}\right)}_{2}\in Z^{km}$. Then we have $∥{\left(e_{{\mathrm{ID}}_{k,i^{\prime }}}\right)}_{1}∥\leq {\bar{\sigma }}_{k}\sqrt{m}$ and ${\left(e_{{\mathrm{ID}}_{k,i^{\prime }}}\right)}_{2}\leq {\bar{\sigma }}_{k}\sqrt{km}$ where ${\bar{\sigma }}_{k}=\sigma_{k}\sqrt{(k+1)m}\omega \left(\sqrt{\log km}\right)$. Since $x_{2,i^{\prime }}=R^{⊤}x_{1,i^{\prime }}$, by Equation (3), $∥x_{2,i^{\prime }}∥=∥R^{⊤}x_{1,i^{\prime }}∥\leq O\left(\sqrt{k}m\right)$

Refer to the formulas in Equations (1) and (2), the error term

(23) $$\begin{array}{cc} \; & |x-e_{{\mathrm{ID}}_{k,i^{\prime }}}^{⊤}\left[\begin{array}{c} x_{1,i^{\prime }}\\ x_{2,i^{\prime }} \end{array}\right]|\\ \leq & \left|x|+\right|e_{{\mathrm{ID}}_{k,i^{\prime }}}^{⊤}\left[\begin{array}{c} x_{1,i^{\prime }}\\ x_{2,i^{\prime }} \end{array}\right]|\\ = & \left|x|+\right|{\left(e_{{\mathrm{ID}}_{k,i^{\prime }}}\right)}_{1}^{⊤}x_{1,i^{\prime }}\left|+\right|{\left(e_{{\mathrm{ID}}_{k,i^{\prime }}}\right)}_{2}^{⊤}x_{2,i^{\prime }}|\\ \leq & q\alpha_{k}\omega \left(\sqrt{\log m}\right)+\frac{1}{2}+{\bar{\sigma }}_{k}\sqrt{m}q\alpha_{k}\omega \left(\sqrt{\log m}\right)+\frac{{\bar{\sigma }}_{k}m}{2}+{\bar{\sigma }}_{k}\sqrt{km}\cdot O\left(\sqrt{k}m\right)\\ \leq & qk^{2}\alpha_{k}\sigma_{k}m\omega \left(\sqrt{\log m}\right)+O\left(k^{3/2}\sigma_{k}m^{2}\right) \end{array}$$

To ensure the correctness of decryption and preform the security proof, for all 1≤k≤d, we need that:

- the error term is less than q/5 with overwhelming probability (w.h.p) (i.e., $\alpha_{k}<{\left[\sigma_{k}m\omega \left(\sqrt{\log m}\right)\right]}^{-1}$, and $q=\Omega (k^{3/2}\sigma_{k}m^{2})$),
- the TrapGen algorithm can operate (i.e., m>3(1+δ)nlogq for some δ>0),
- the Leftover Hash Lemma can be applied to security proof (i.e., m>(n+1)logq+ω(logn)),
- the SampleBasisLeft and SampleBasisRight algorithm can operate (i.e., $\sigma_{k}>\sigma_{TG}\sqrt{m}\omega \left(\sqrt{\log m}\right)$ where $\sigma_{TG}=O\left(\sqrt{n\log q}\right)$), and
- the Regev’s LWE reduction applies(i.e., $q>2\sqrt{n}/\alpha$).

### 4.3. Security

**Theorem** **3.** *If the $\left(Z_{q},n,{\bar{\Psi }}_{\alpha }\right)$-LWE assumption holds, the above HIB-DRE scheme is IND-ID-CPA secure.*

**Proof.**  Let A be a probabilistic polynomial-time(PPT) adversary that can break our IB-DRE scheme with advantage ε>0. Then there exists a reduction that solves the $\left(Z_{q},n,{\bar{\Psi }}_{\alpha }\right)$-LWE problem with an negligible advantage. Let Q=Q(n) is the upper bound of the number of KeyGen queries and $I=\{\left({\mathrm{ID}}_{k,1}^{*},{\mathrm{ID}}_{k,2}^{*}\right),{\left({\mathrm{ID}}_{k,1}^{յ},{\mathrm{ID}}_{k,2}^{յ}\right)}_{j\in [Q]}\}$ denotes the challenge IDs. Different from $Q\leq \frac{q}{2}$ in [11], here we let $Q\leq \frac{n^{c}}{4}-1$ where c=c(n). We show the security via the following games. In each game, we define a value r∈{0,1} and let $W_{i}$ denote the event that the adversary correctly guessed the challenge bit, i.e., the challenger output $r^{\prime }=r$ in ${\mathrm{Game}}_{i}$. $|Pr\left[W_{i}\right]-\frac{1}{2}|$ is the adversary’s advantage.

${\mathrm{Game}}_{0}$. This is the real IND-ID-CPA game between an adversary A and the challenger. So we have

$$|\mathrm{Pr}\left[W_{0}\right]-\frac{1}{2}|=|\mathrm{Pr}\left[r^{\prime }=r\right]-\frac{1}{2}|=\varepsilon .$$

${\mathrm{Game}}_{1}$. The same as ${\mathrm{Game}}_{1}$ in Section 3.3, in this game we also add an abort event at the end of the game. The challenger chooses $y=\{y_{0},{\left(y_{i,j}^{k}\right)}_{\left(i,j)\in [p,l],k\in [d\right]}\}$ where $y_{0}\leftarrow \left[-\left(n+1\right){\left(pn^{c}\right)}^{p},0\right]$ and $y_{i,j}^{k}\leftarrow \left[1,pn^{c}\right]$. Let $h_{{\mathrm{id}}_{k}}=y_{0}+\sum_{\left(j_{1},\cdots ,j_{p}\right)\in F\left({\mathrm{id}}_{k}\right)}y_{i,j_{1}}^{k}\cdots y_{i,j_{p}}^{k}$ and *H* be a function such that

$$H\left({\mathrm{ID}}_{k}\right)=h_{{\mathrm{id}}_{1}}|h_{{\mathrm{id}}_{2}}\left|\cdots \right|h_{{\mathrm{id}}_{k}}.$$

For the challenge IDs, the challenger checks whether $H({\mathrm{ID}}_{k,i^{\prime }}^{*})=0$ and $H({\mathrm{ID}}_{k,i^{\prime }}^{յ})\neq 0$ where $i^{\prime }\in \left\{1,2\right\}$. If they do not hold, the game aborts. Otherwise, the challenger outputs $r^{\prime }=r$.

Different to the Lemma 8, the probability η(I) satisfies

$$\frac{k}{{\left(n+1\right)}^{2}}\cdot {\left(\frac{1}{pn^{c}}\right)}^{2p}\cdot {\left(1-\frac{Q}{n^{c}}\right)}^{2}\leq \eta \left(I\right)\leq \frac{k}{{\left(n+1\right)}^{2}}\cdot {\left(\frac{1}{pn^{c}}\right)}^{2p}.$$

Then by $|\mathrm{Pr}\left[W_{1}\right]-\frac{1}{2}|=\eta_{\min}\cdot \varepsilon -\left(\eta_{\max}-\eta_{\min}\right)/2$, we have

$$|\mathrm{Pr}\left[W_{1}\right]-\frac{1}{2}|\geq \frac{k}{{\left(n+1\right)}^{2}}\cdot {\left(\frac{1}{pn^{c}}\right)}^{2p}\cdot \left(\varepsilon -\frac{2Q}{n^{c}}\right)$$

${\mathrm{Game}}_{2}$. In this game, we change the way $A_{i,j}^{k}$, ${\bar{A}}_{i,j}^{k}$, $B_{0}$ are generated. The challenger firstly chooses $y=\{y_{0},{\left(y_{i,j}^{k}\right)}_{\left(i,j)\in [p,l],k\in [d\right]}\}$, $y^{{}^{\prime }}=\left\{y_{0},{\left({\bar{y}}_{i,j}^{k}\right)}_{\left(i,j)\in [p,l],k\in [d\right]}\right\}$ as ${\mathrm{Game}}_{1}$ and chooses three matrices $R_{0},R_{i,j}^{k},{\bar{R}}_{i,j}^{k}\in {\left\{-1,1\right\}}^{m\times m}$. Compute

(24) $$B_{0}=A_{0}R_{0}+y_{0}G$$

(25) $$A_{i,j}^{k}=A_{0}R_{i,j}^{k}+y_{i,j}^{k}G$$

(26) $${\bar{A}}_{i,j}^{k}=A_{0}{\bar{R}}_{i,j}^{k}+{\bar{y}}_{i,j}^{k}G$$

Based on Lemma 6, the distribution (A, $A_{0}R_{0}+y_{0}G$, $A_{0}R_{i,j}^{k}+y_{i,j}^{k}G$, $A_{0}{\bar{R}}_{i,j}^{k}+{\bar{y}}_{i,j}^{k}G$) is negl(n)-close to the distribution (A, $B_{0}$, $A_{i,j}^{k}$, ${\bar{A}}_{i,j}^{k}$). Therefore, we have

$$|\mathrm{Pr}\left[W_{1}\right]-\mathrm{Pr}\left[W_{2}\right]|=\mathrm{negl}\left(n\right).$$

Before the next game, let

$${\hat{R}}_{{\mathrm{ID}}_{k,1}}=R_{0}+\sum_{\left(j_{1},\cdots ,j_{p}\right)\in F\left({\mathrm{ID}}_{k,1}\right)}{\mathrm{TrapEval}}_{p}\left(R_{1,j_{1}}^{k},\cdots ,R_{p,j_{p}}^{k}\right),$$

$${\hat{R}}_{{\mathrm{ID}}_{k,2}}=R_{0}+\sum_{\left(j_{1},\cdots ,j_{p}\right)\in F\left({\mathrm{ID}}_{k,2}\right)}{\mathrm{TrapEval}}_{p}\left({\bar{R}}_{1,j_{1}}^{k},\cdots ,{\bar{R}}_{p,j_{p}}^{k}\right).$$

Based on Lemma 3, we have

(27) $$\begin{array}{c} ∥{\hat{R}}_{{\mathrm{ID}}_{k,1}}∥=∥R_{0}+{\hat{R}}_{{\mathrm{ID}}_{k,1}}∥\leq ∥R_{0}∥+\sum_{\left(j_{1},\cdots ,j_{p}\right)\in F\left({\mathrm{ID}}_{k,1}\right)}∥{\mathrm{TrapEval}}_{p}\left(R_{1,j_{1}}^{k},\cdots ,R_{p,j_{p}}^{k}\right)∥\\ \leq m+nmp{\left(pn^{c}\right)}^{p-1}=m\left(1+p^{p}n^{cp-c+1}\right). \end{array}$$

$∥{\hat{R}}_{{\mathrm{ID}}_{k,2}}∥$ is the same as $∥{\hat{R}}_{{\mathrm{ID}}_{k,1}}∥$.

Then define $R_{{\mathrm{ID}}_{k,i^{\prime }}}={\hat{R}}_{{\mathrm{ID}}_{1,i^{\prime }}}\left|\cdots \right|{\hat{R}}_{{\mathrm{ID}}_{k,i^{\prime }}}\in Z_{q}^{m\times km}$ where $i^{\prime }\in \left\{1,2\right\}$. We have $∥R_{{\mathrm{ID}}_{k,i^{\prime }}}∥\leq km\left(1+p^{p}n^{cp-c+1}\right)$.

${\mathrm{Game}}_{3}$. In this game, we change the way $A_{0}$ is generated. The challenger chooses a random matrix $A_{0}\in Z_{q}^{n\times m}$ instead of using the TrapGen algorithm. For the secret key queries, the challenger responds by the SampleRight instead of SampleRight. By the definition of $R_{{\mathrm{ID}}_{k}}$, we have

$$H\left({\mathrm{ID}}_{k,1}\right)=A_{0}\cdot \left({\hat{R}}_{{\mathrm{ID}}_{1,1}}+h_{{\mathrm{id}}_{1,1}}G\right)\left|\cdots \right|A_{0}\cdot \left({\hat{R}}_{{\mathrm{ID}}_{k,1}}+h_{{\mathrm{id}}_{k,1}}G\right),$$

$$H\left({\mathrm{ID}}_{k,2}\right)=A_{0}\cdot \left({\hat{R}}_{{\mathrm{ID}}_{1,2}}+h_{{\mathrm{id}}_{1,2}}G\right)\left|\cdots \right|A_{0}\cdot \left({\hat{R}}_{{\mathrm{ID}}_{k,2}}+h_{{\mathrm{id}}_{k,2}}G\right).$$

Therefore for $i^{\prime }\in \left\{1,2\right\}$,

(28) $$F_{{\mathrm{ID}}_{k,i^{\prime }}}=A_{0}\left|H\left({\mathrm{ID}}_{k,i^{\prime }}\right)=A_{0}\right|A_{0}\left(R_{{\mathrm{ID}}_{k,i^{\prime }}}+G_{{\mathrm{ID}}_{k,i^{\prime }}}\right)$$

where $R_{{\mathrm{ID}}_{k,i^{\prime }}}={\hat{R}}_{{\mathrm{ID}}_{1,i^{\prime }}}\left|\cdots \right|{\hat{R}}_{{\mathrm{ID}}_{k,i^{\prime }}}$ and $G_{{\mathrm{ID}}_{k,i^{\prime }}}=h_{{\mathrm{id}}_{1,i^{\prime }}}G\left|\cdots \right|h_{{\mathrm{id}}_{1,i^{\prime }}}G$.

If $H({\mathrm{ID}}_{k,1})=0$ or $H({\mathrm{ID}}_{k,2})=0$, the challenger aborts and returns a random bit. Otherwise, it returns $E_{1}$ and $E_{2}$ to A where

$$E_{1}\leftarrow \mathrm{SampleBasisRight}\left(A_{0},G_{{\mathrm{ID}}_{k,1}},R_{{\mathrm{ID}}_{k,1}},T_{G},\sigma_{k}\right)$$

$$E_{2}\leftarrow \mathrm{SampleBasisRight}\left(A_{0},G_{{\mathrm{ID}}_{k,2}},R_{{\mathrm{ID}}_{k,2}},T_{G},\sigma_{k}\right)$$

In particular, the challenger checks if the challenge identity $\left({\mathrm{ID}}_{k,1}^{*},{\mathrm{ID}}_{k,2}^{*}\right)$ satisfies $H({\mathrm{ID}}_{k,1}^{*})=0$ and $H({\mathrm{ID}}_{k,2}^{*})=0$. If not, the game aborts as in ${\mathrm{Game}}_{1}$.

Since in the adversary’s view, ${\mathrm{Game}}_{2}$ and ${\mathrm{Game}}_{3}$ are identical (the public parameters, abort conditions, responses to private key queries and the challenge ciphertext). The advantage of the adversary A is identical to ${\mathrm{Game}}_{2}$, i.e.,

$$|\mathrm{Pr}\left[W_{2}\right]-\mathrm{Pr}\left[W_{3}\right]|=\mathrm{negl}\left(n\right).$$

${\mathrm{Game}}_{4}$. As we know, the ciphertext space is $C\in Z_{q}\times Z_{q}^{(k+1)m}\times Z_{q}^{(k+1)m}$. In this game, the challenger set the ciphertext as $c^{*}=\left\{c_{0}^{*},c_{1}^{*},c_{2}^{*}\right\}$ which is uniformly random in $Z_{q}\times Z_{q}^{(k+1)m}\times Z_{q}^{(k+1)m}$ in the challenge phase. And the advantage of the adversary A is 0. As shown in Lemma 10, assuming $\left(Z_{q},n,{\bar{\Psi }}_{\alpha }\right)$-LWE holds, ${\mathrm{Game}}_{3}$ and ${\mathrm{Game}}_{4}$ are computationally indistinguishable, i.e., $|\mathrm{Pr}\left[W_{3}\right]-\mathrm{Pr}\left[W_{4}\right]|=\mathrm{negl}\left(n\right).$ ☐

**Lemma** **10.** *For any PPT adversary A, there exists a challenger B such that*

$$|\mathrm{Pr}\left[W_{3}\right]-\mathrm{Pr}\left[W_{4}\right]|\leq \mathrm{LWE}-Adv\left(B\right).$$

**Proof of Lemma** **10.** Suppose A has a non-negligible advantage in distinguishing ${\mathrm{Game}}_{4}$ and ${\mathrm{Game}}_{5}$. We use A to construct an LWE algorithm denoted B.

Recall from Definition 4 that an LWE problem instance is provided as a sampling oracle O which is either a truly random sampler $O_{s}^{{}^{\prime }}$ or a noisy pseudo-random sampler $O_{s}$ for a secret $s\in Z_{q}^{n}$. The challenger B uses the adversary A to distinguish which the sampler it is given, and proceeds as follows:

**Instance**. The challenger B requests from O to obtain (m+1) LWE samples that we denote as:

$$\left\{\left(w_{0},v_{0}\right),\left(w_{1},v_{1}\right),\left(w_{2},v_{2}\right),\ldots ,\left(w_{m},v_{m}\right)\right\}\in \left(Z_{q}^{n}\times Z_{q}\right)$$

**Setup**. The challenger B constructs the public parameters pp as follows:

(1) Construct a matrix $A_{0}\in Z_{q}^{n\times m}$ by assembling *m* LWE samples such that $A_{0}=\left(w_{1},w_{2},\ldots ,w_{m}\right)$, and let $u=w_{0}$.
(2) Choose y as in ${\mathrm{Game}}_{1}$ and constructs the remainder of the public parameters as in ${\mathrm{Game}}_{2}$.
(3) Send the $pp=\{A_{0},{\left(A_{i,j}^{k},{\bar{A}}_{i,j}^{k}\right)}_{\left(i,j)\in [p,l],k\in [d\right]},B_{0},u\}$ to A.

**Queries**. A makes secret key query. The challenger B computes and checks if $H({\mathrm{ID}}_{k})=0$. If it holds, it aborts. Otherwise it generate the secret key for A as in ${\mathrm{Game}}_{3}$.

**Challenge**. A sends a message bit $\mu^{*}\in \left\{0,1\right\}$ and the target identities $(\left({\mathrm{ID}}_{k,1}^{*},{\mathrm{ID}}_{k,2}^{*}\right)$ to B. The challenger B constructs $v^{*}={\left(v_{1},v_{2},\cdots ,v_{m}\right)}^{⊤}$ where $v_{0},v_{1},\cdots ,v_{m}\in Z_{q}$ is the LWE samples. Let $R_{{\mathrm{ID}}_{k,1}^{*}}={\hat{R}}_{{\mathrm{ID}}_{1,1}^{*}}\left|\cdots \right|{\hat{R}}_{{\mathrm{ID}}_{k,1}^{*}}\in Z_{q}^{m\times km}$ and $R_{{\mathrm{ID}}_{k,2}^{*}}={\hat{R}}_{{\mathrm{ID}}_{1,2}^{*}}\left|\cdots \right|{\hat{R}}_{{\mathrm{ID}}_{k,2}^{*}}\in Z_{q}^{m\times km}$. Then the challenge ciphertext

$$c_{0}^{*}=v_{0}+\mu^{*}\left\lfloor q/2\right\rfloor ,$$

$$c_{1}^{*}=\left[\begin{array}{c} v^{*}\\ R_{{\mathrm{ID}}_{k,1}^{*}}^{⊤}v^{*} \end{array}\right],$$

$$c_{2}^{*}=\left[\begin{array}{c} v^{*}\\ R_{{\mathrm{ID}}_{k,2}^{*}}^{⊤}v^{*} \end{array}\right].$$

B sends the challenge ciphertext $c^{*}=\left\{c_{0}^{*},c_{1}^{*},c_{2}^{*}\right\}$ to A.

Note that when $O=O_{s}$, the ciphertext is valid.(We just argue only when no abort happens). Since $H({\mathrm{ID}}_{k,1}^{*})=0$ and $H({\mathrm{ID}}_{k,2}^{*})=0$, we have $F_{{\mathrm{ID}}_{k,1}^{*}}=A_{0}\left|H\left({\mathrm{ID}}_{k,1}^{*}\right)=A_{0}\right|A_{0}R_{{\mathrm{ID}}_{k,1}^{*}}$. The same as $F_{{\mathrm{ID}}_{k,1}^{*}}$, we have $F_{{\mathrm{ID}}_{k,2}^{*}}=A_{0}|A_{0}R_{{\mathrm{ID}}_{k,2}^{*}}$. By definition of $O_{s}$, we know $v^{*}=A_{0}^{⊤}s+x$. Then we have

(29) $$c_{0}^{*}=v_{0}+\mu^{*}\left\lfloor q/2\right\rfloor =\left(u^{⊤}s+x_{0}\right)+\mu^{*}\left\lfloor q/2\right\rfloor$$

(30) $$\begin{array}{cc} c_{1}^{*} & =\left[\begin{array}{c} v^{*}\\ R_{{\mathrm{ID}}_{k,1}^{*}}^{⊤}v^{*} \end{array}\right]=\left[\begin{array}{c} A_{0}^{⊤}s+x\\ R_{{\mathrm{ID}}_{k,1}^{*}}^{⊤}\left(A_{0}^{⊤}s+x\right) \end{array}\right]\\ & =\left[\begin{array}{c} A_{0}^{⊤}s+x\\ A_{0}R_{{\mathrm{ID}}_{k,1}^{*}}{]}^{⊤}s+R_{{\mathrm{ID}}_{k,1}^{*}}^{⊤}x \end{array}\right]=F_{{\mathrm{ID}}_{k,1}^{*}}^{⊤}s+\left[\begin{array}{c} x\\ R_{{\mathrm{ID}}_{k,1}^{*}}^{⊤}x \end{array}\right] \end{array}$$

The same as $c_{1}^{*}$, $c_{2}^{*}$ is also valid. It is also similar to the ${\mathrm{Game}}_{3}$.

When $O=O_{s}^{{}^{\prime }}$, $v_{0}\in Z_{q}$ and $v^{*}\in Z_{q}^{m}$ are all uniform. Therefore $c_{0}^{*},c_{1}^{*},c_{2}^{*}$ are uniform in $\in Z_{q}\times Z_{q}^{(k+1)m}\times Z_{q}^{(k+1)m}$ by the Lemma 6.

**Guess**. After being allowed to make additional secret key queries, A guesses if it is interacting with a ${\mathrm{Game}}_{4}$ or ${\mathrm{Game}}_{5}$ challenger. B output A’s guess as the answer to the LWE challenge it is trying to solve. Therefore we have $\mathrm{LWE}-Adv\left(B\right)=|\mathrm{Pr}\left[W_{3}\right]-\mathrm{Pr}\left[W_{4}\right]|$. ☐

**Remark** **1.** *Note that, as in [10,11], the two schemes also can encrypt multiple message bits. To encrypt n-bits message we need to include n vectors *$u_{1},u_{2},\cdots ,u_{n}$*in the public parameters*pp*. Let*$U=(u_{1},u_{2},\cdots ,u_{n})$*and replace the vector*u*with*U*. Then taking each element of*U*as input in the KeyGen phase in the IB-DRE scheme or the Decrypt phase in the HIB-DRE scheme such that*$e_{{\mathrm{id}}_{i^{\prime }}}\leftarrow SampleLeft\left(A_{0},H\left({\mathrm{id}}_{i^{\prime }}\right),u_{i},T_{A_{0}}\right)$*,*$e_{{\mathrm{ID}}_{k,i^{\prime }}}\leftarrow SamplePre\left(F_{{\mathrm{ID}}_{k,i^{\prime }}},sk_{{\mathrm{ID}}_{k,i^{\prime }}},u_{i},{\bar{\sigma }}_{k}\right)$*. Moreover, replace the ciphertext*$c_{0}$*with*$c_{0}=U^{⊤}s+x+\mu \left\lfloor q/2\right\rfloor \in Z_{q}^{n}$*. The proof of security is basically unchanged, except that in the **Instance** phase*B makes m+n *times queries of the LWE oracle instead of* m+1 *times.*

## 5. Performance Analysis

Here we firstly give the comparison between lattice-based IB-DRE scheme and other related IB-DRE schemes which are based on bilinear maps. As shown in Table 1, compared to [4], our scheme and [20] can resist quantum attack due to the fact that our scheme and [20] are based on the LWE problem on lattices which is proved by Regev [9] to resist quantum computing attack, but [4] is based on the decisional bilinear Diffie–Hellman (DBDH) problem which can not resist quantum computing attack. In addition, [4,20] and our scheme are all proved to be CPA secure. To lighten the pressure of KGC, we extend our scheme to the hierarchical scenario, but [4,20] can not support hierarchical scenario.

Next we give the comparison of storage cost, communication cost and computational cost between our construction and the first lattice-based IB-DRE scheme (next we use how Zhang18 denotes it).

**Figure 1:**
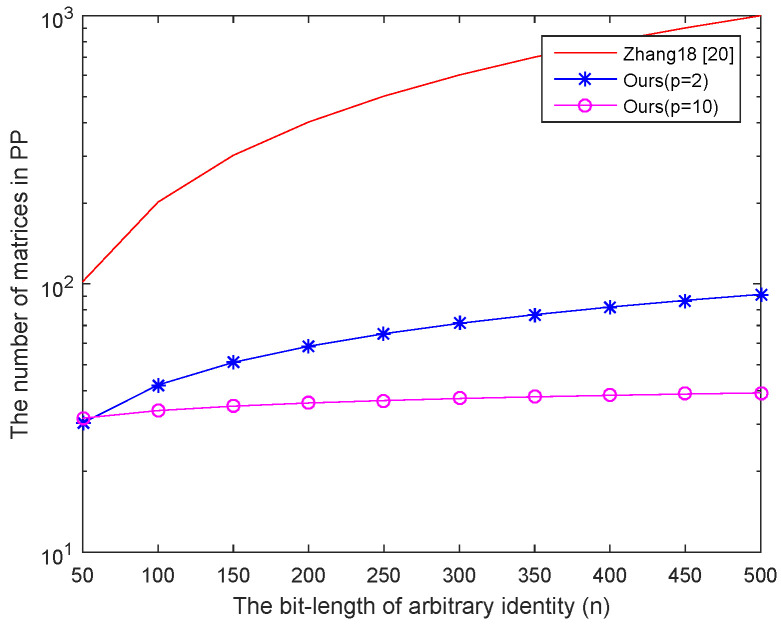
The number of the matrices in public parameters.

**Storage costs analysis**. Here we give the comparison of storage costs between our construction and the first lattice-based IB-DRE scheme (next we use how Zhang18 denotes it). In Zhang18, the authors propose an adaptively secure IB-DRE scheme based on the LWE problem. In their scheme, to achieve the adaptively-ID secure, they generate 2n matrices in the Setup phase by using the same method of [11]. The size of public parameters is $O(n^{3}\log q)$ which lead to a high storage overhead. As shown in Table 2, the suggested lattice dimension *m* in our scheme is smaller than [20] under the same adaptively secure model. Since *p* is a flexible constant which can directly affect the pp size, we give the comparison results when *p* takes different values. Since we introduce an injective map function in our construction, the public parameters size can be reduced from 2n+2 matrices to $2pn^{\frac{1}{p}}+2$ matrices where p(≥2) is a flexible constant. Namely, the storage cost of public parameters pp is reduced from $O(n^{3}\log q)$ to $O(n^{2+\frac{1}{p}}\log q)$. Moreover, the user’s secret key in our scheme is smaller than [20] and the ciphertext is equal to [20]. Figure 1 shows that when p=2 and n=284 according to the suggestion given by Micciancio et al. in [22], the size of public parameters in our scheme is reduced by merely 88% of Zhang et al.’s method. Not to mention when p>2 or n>284. In addition, from the Figure 1, with the increasing of the bit-length of identity, the size of public parameters in our scheme and [20] are also increase. It is still smaller than [20]. In addition, in our scheme, when p=2, the size of the public parameters in our scheme achieves the largest of our scheme, and it is 12% of [20]. Not to mention when p>2.

**Communication costs analysis**. There are four algorithms in our scheme and in Zhang18 [20]. During the operation of the algorithm, three transmissions of public parameters, two transmissions of ciphertext and at least two transmissions of user’s secret key are required. According to the comparison results of Table 2 and Figure 1, under the same transmission bandwidth, it is obvious that communication costs of our public parameters and user’s secret key are faster than in Zhang18 [20]. Communication cost of the ciphertext is equal to Zhang18 [20].

**Computational costs analysis**. As shown in Table 3, we compared our scheme with Zhang18 on computational costs. The computational cost of encryption in our construction is equal to the related lattice-based IB-DRE scheme in [20]. As for the computational cost of KeyGen, in [20] the user’s secret key is a 2m×n matrix while it is a 2m dimensions vector in our scheme. Thus, the computational cost of KeyGen in [20] is larger than our scheme. Due to the fact that the size of user’s secret in [20] is *n* times larger than us, the computational cost is also larger than us.

## 6. Conclusions

Different from the standard cryptographic primitive of public key encryption by which a ciphertext can usually be decrypted by the private-key holders only, dual receiver encryption enables a ciphertext to be decrypted to the same plaintext by two different but dual receivers. In this paper, we propose two more efficient constructions of (hierarchical) identity-based dual receiver encryption schemes from lattices which can resist quantum attack. By combining an injective map and a homomorphic computation technique, the size of public parameters is remarkably reduced from 2n+2 matrices to $2pn^{\frac{1}{p}}+2$ matrices where p(≥2) is a flexible constant. Compared to the only related work—Zhang18, about 88% = (1–12%) storage cost is saved by using our method. Under the intractability assumption of the learning with errors problem over lattices, our proposal was proved to be semantically secure against adaptively chosen identity and plaintext attacks.

## Figures and Tables

**Table 1:** The comparison between lattice-based identity-based device receiver encryption (IB-DRE) and bilinear maps-based IB-DRE.

Scheme	Security Assumption	Resistance to Quantum Attack	CPA/CCA	Hierarchical
[4]	DBDH	No	CPA	No
[20]	LWE	Yes	CPA	No
Ours	LWE	Yes	CPA	Yes

**Table 2:** The comparison between our scheme and related lattice-based scheme.

Schemes	Lattice Dimension *m*	*pp*	${\mathrm{sk}}_{\mathrm{id}}$	Ciphertext	1/α for LWE Assumption	Selective/Adaptive
Zhang18 [20]	6nlogq	$O(n^{3}\log q)$	2mnlogq	$O(n^{3}\log q)$	Fixed poly(n)	Adaptive
Ours IB-DRE	3nlogq	$O(n^{2+\frac{1}{p}}\log q)$	2mlogq	$O(n^{3}\log q)$	All poly(n)	Adaptive
		p(≥2)				

Fixed poly(*n*): a scheme is proven secure under the LWE assumption with 1/*α* being some fixed polynomial (e.g.,*n*^3^). All poly(*n*): a scheme is proven secure under the LWE assumption with 1/*α* being all polynomial.

**Table 3:** The comparison of computational cost.

Scheme	KeyGen	Encryption	Decryption
Zhang18 [20]	$O(n^{2}m^{2})$	$O(n^{2}m)$	$O(nm^{2})$
Ours	$O(nm^{2})$	$O(n^{2}m)$	$O(m^{2})$
